# Isolation and identification of uric acid-dependent *Aciduricibacillus chroicocephali* gen. nov., sp. nov. from seagull feces and implications for hyperuricemia treatment

**DOI:** 10.1128/msphere.00025-24

**Published:** 2024-05-30

**Authors:** Wenxuan Liu, Fulong Nan, Fengjun Liu, Xiaoli Yang, Zonghui Li, Shasha Jiang, Xianjuan Zhang, Jun Li, Meng Yu, Yunyang Wang, Bin Wang

**Affiliations:** 1 Department of Pathogenic Biology, Department of Special Medicine, School of Basic Medicine, Qingdao University, Qingdao, China; 2 Department of Clinical Laboratory, The Affiliated Hospital of Qingdao University, Qingdao, China; 3 Department of Endocrinology and Metabolism, The Affiliated Hospital of Qingdao University, Qingdao, China; University of Michigan-Ann Arbor, Ann Arbor, Michigan, USA

**Keywords:** *Aciduricibacillus chroicocephali*, uric acid, urate oxidase, hyperuricemia, urate-lowering treatment

## Abstract

Hyperuricemia has become the second most prevalent metabolic disease after diabetes, but the limitations of urate-lowering treatment (ULT) drugs and patient nonadherence make ULT far less successful. Thus, more ULT approaches urgently need to be explored. Uric acid-degrading bacteria have potential application value in ULT. In this study, we isolated 44XB^T^, a uric acid-degrading bacterium, from black-headed gull (*Chroicocephalus ridibundus*) feces. Using a polyphasic taxonomic approach, strain 44XB^T^ was identified as a novel genus within the family *Bacillaceae*; subsequently, the name *Aciduricibacillus chroicocephali* was proposed. Strain 44XB^T^ had a unique uric acid-dependent phenotype and utilized uric acid and allantoin as the sole carbon and nitrogen sources, but not common carbon sources or complex media. In the genome, multiple copies of genes involved in uric acid metabolic pathway (*pucL*, *pucM*, *uraD*, and *allB*) were found. Six copies of *pucL* (encoding urate oxidase) were detected. Of these, five *pucL* copies were in a tandem arrangement and shared 70.42%–99.70% amino acid identity. *In vivo* experiments revealed that 44XB^T^ reduced serum uric acid levels and attenuated kidney damage in hyperuricemic mice through uric acid catalysis in the gut and gut microbiota remodeling. In conclusion, our findings discover a strain for studying bacterial uric acid metabolism and may provide valuable insights into ULT.

**IMPORTANCE:**

The increasing disease burden of hyperuricemia highlights the need for new therapeutic drugs and treatment strategies. Our study describes the developmental and application values of natural uric acid-degrading bacteria found in the gut of birds and broadened the source of bacteria with potential therapeutic value. Furthermore, the special physiology characteristics and genomic features of strain 44XB^T^ are valuable for further study.

## INTRODUCTION

The loss of urate oxidase activity provided a certain evolutionary advantage for early *Homo sapiens*, but with changes in dietary structures and psychosocial influences, circulating uric acid has increased significantly, bringing obvious detrimental effects. Hyperuricemia has become the second most prevalent metabolic disease after diabetes, with gout as the major complication, and is associated with metabolic and cardiovascular comorbidities such as chronic kidney disease (CKD), hypertension, and type II diabetes (1). The prevalence rates of hyperuricemia and gout have increased continuously over the past two decades, with gout affecting more than 50 million people globally, making it a growing public health problem (2). The main factors contributing to hyperuricemia involve the overproduction and underexcretion of uric acid, which are the main strategies for the treatment of hyperuricemia. Commonly used urate-lowering treatment (ULT) drugs include xanthine oxidase inhibitors (e.g., allopurinol and febuxostat), uricosuric drugs (e.g., benzbromarone), and recombinant uricase drugs (e.g., pegloticase). However, the effectiveness of ULT is considered far less successful due to the limitations of ULT drugs and patient nonadherence (3, 4). Therefore, there is a pressing need to explore more ULT approaches.

The dysbiosis of the gut microbiota is associated with hyperuricemia, and microbial remediation is becoming a strategy of increasing interest for the treatment of hyperuricemia (3, 5). For example, the marketed probiotics *Lactobacillus gasseri* PA-3 and *Limosilactobacillus fermentum* GR-3 positively impact hyperuricemia by degrading purines or uric acid and regulating gut microbiota (6, 7). However, the strains successfully used for treatment are mainly concentrated in a limited number of traditional probiotic genera (e.g., *Lactobacillus*, *Lacticaseibacillus*, and *Limosilactobacillus*), and most of them are isolated from fermented foods such as yogurt and cheese; few are isolated from other sources (7–9). Normally, approximately two-thirds of uric acid is excreted through the kidneys via uric acid transporters, and the remaining third is transported into the gut lumen to be metabolized by intestinal bacteria or excreted in feces (10). In patients with CKD, due to renal insufficiency, uric acid excretion through the kidneys is impaired, and up to 50%–70% of uric acid is excreted through the gut (11, 12). The importance of the gut in maintaining uric acid homeostasis highlights the potential of targeting gut uric acid degradation as a possible ULT strategy. Bacteria participate in uric acid metabolism through two main pathways: one is the classical metabolic pathway from uric acid to allantoin catalyzed by urate oxidase in aerobic bacteria, while the other is a recently discovered pathway found in anaerobic bacteria, where uric acid is catalyzed into xanthine and short-chain fatty acids (13, 14). Urate oxidase has value in uric acid degradation in the gut although the gut is relatively anaerobic. ALLN-346, an orally administered recombinant urate oxidase optimized for activity and proteolytic stability, has demonstrated effects has demonstrated effects in urate oxidase deficient mice, and patients with CKD (11). Another example is the engineered urate oxidase-producing *Escherichia coli* strain and has shown positive therapeutic effects in the hyperuricemic mouse (15, 16). Compared with oral enzymes and engineered bacteria, the search for new, natural, efficient, and relatively safe uric acid-degrading bacteria holds promise in enhancing the cost-effectiveness of ULT and broadening the bacterial sources for treating hyperuricemia.

In this study, we isolated and identified a novel uric acid-degrading genus that showed uric acid-dependent metabolic characteristics and validated its urate-lowering effects *in vivo*. Our findings may provide valuable insights into hyperuricemia treatment.

## RESULTS

### Isolation and phenotypic characterization of 44XB^T^

**Fig 1:**
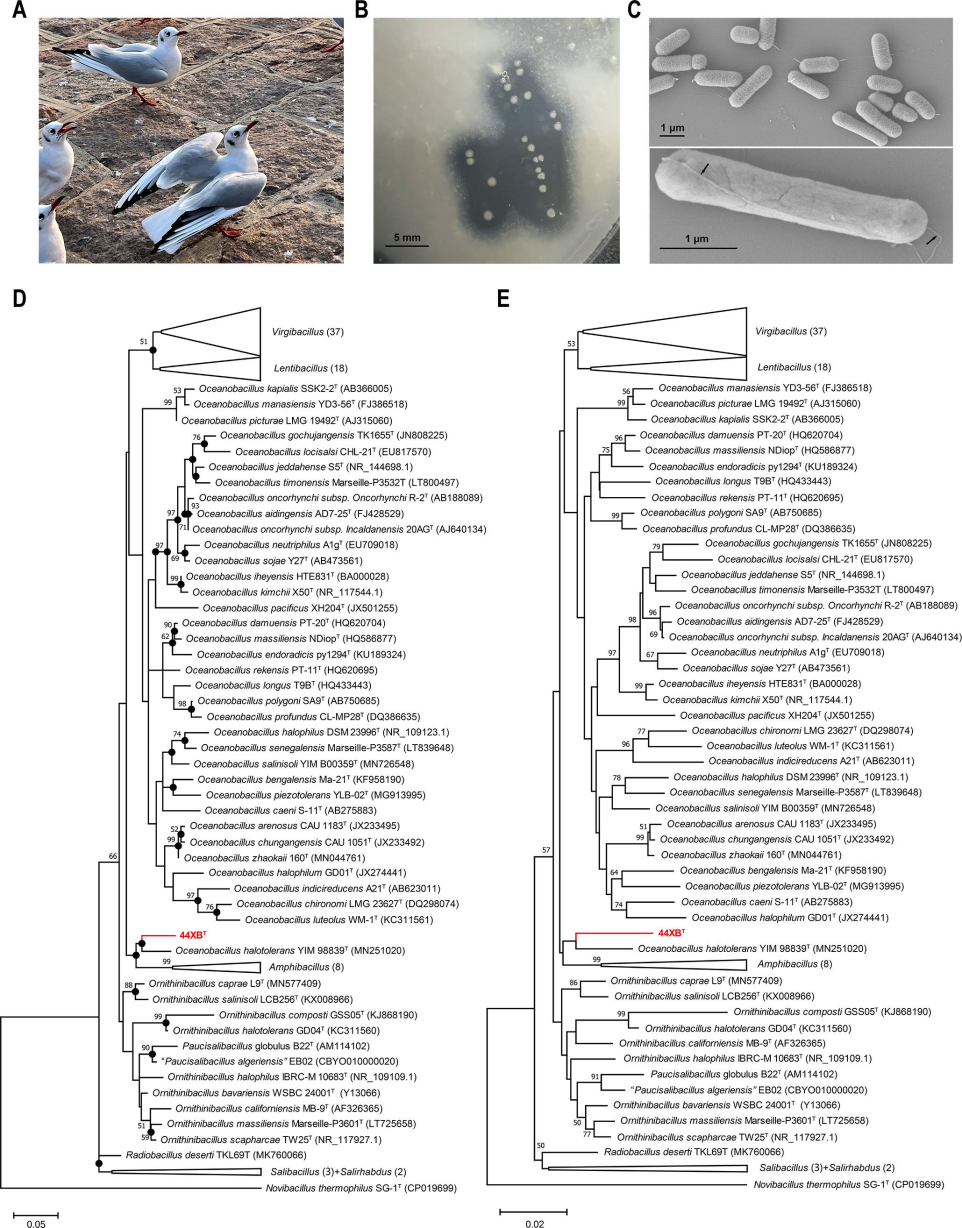
Colonies, morphology, and 16S rRNA based phylogenetic trees of *A. chroicocephali* 44XB^T^. (**A**) Seagulls in the Nanjiang Wharf. (**B**) Colony morphology of strain 44XB^T^ after 2 days of incubation at 37°C on UA medium. (**C**) SEM image of strain 44XB^T^ and flagella. Cells were collected after 8 h of culture in UA medium. The scale bar is 1 µm. (**D**) The 16S rRNA maximum-likelihood tree of strain 44XB^T^. Bootstrap values greater than 50% are shown at branch points. Filled circles indicate that the corresponding nodes are reproduced in the neighbor-joining tree. Bar, 0.05 substitutions per nucleotide position. (**E**) The 16S rRNA neighbor-joining tree of strain 44XB^T^. Bar, 0.02 substitutions per nucleotide position.

To screen the uric acid-degrading bacteria, black-headed gull fecal samples were collected at the Qingdao Nanjiang Wharf, and a bacterium with observable urate oxidase activity was isolated, named 44XB^T^ (Fig. 1A). 44XB^T^ formed creamy-white, smooth, and circular colonies with a diameter of approximately 1–1.5 mm on uric acid medium (UA medium), and the transparent zones representing the active degradation of the uric acid contained in the medium by colonial bacteria were clearly observable (Fig. 1B). The novel isolate was gram-positive, strictly aerobic, motile, and rod-shaped, with dimensions of approximately 1.5–2.5 µm in length and 0.6–0.7 µm in width. Scanning electron microscope (SEM) imaging revealed the bacteria’s flagella (Fig. 1C).

Strain 44XB^T^ produced urate oxidase, and the activity of total cell lysate was determined to be 5.14 U per mg protein when the OD_600_ reached 1.2 in UA medium. In minimal media with different carbon and nitrogen sources, strain 44XB^T^ used uric acid and allantoin as the sole carbon and nitrogen sources, but not common carbon sources or amino acids, including glucose, pyruvate, serine, glycine, and among others (Table S1). No detectable growth was observed on complex media, including LB medium, NA, BHI medium, or blood agar, indicating that 44XB^T^ was dependent on uric acid (including the downstream metabolite allantoin) (Fig. S1). In physiological and biochemical tests, 44XB^T^ was catalase and oxidase positive. In the API 20NE test, no positive reactions were observed (Table S2). In the API ZYM test, 44XB^T^ resulted in positive reactions for alkaline phosphatase, esterase lipase (C8), and acid phosphatase (Table S3). In the Biolog GENIII test, no substrate was strongly utilized, which agreed with the results of our carbon source utilization tests (Table S4).

In the chemotaxonomic characterization, the major respiratory quinone of 44XB^T^ was menaquinone-7 (MK-7), which was similar to those of the five reference strains (Fig. S2A through C). The main whole-cell fatty acids (>10% of the total) were anteiso-C_15:0_ (31.83%), iso-C_15:0_ (19.79%), iso-C_14:0_ (11.34%), C_16:0_ (10.76%), and iso-C_16:0_ (10.50%) (Table S5). The identified polar lipids were phosphatidylglycerol (PG), diphosphatidylglycerol (DPG), phosphatidylethanolamine (PE), and three unidentified aminophospholipids (APLs) (Fig. S2D through G). Compared with the five reference strains, the main whole-cell fatty acids and polar lipids of 44XB^T^ contained characteristic C_16:0_ and PE, respectively, which could be well distinguished from the reference strains. Polar lipids, major fatty acids, genome size, and DNA G + C content clearly differentiated strain 44XB^T^ from the reference strains (Table 1).

**TABLE 1:** Differential phenotypic, physiological, and biochemical characteristics of *A. chroicocephali* 44XB^T^ and the 5 reference strains*^a,b^*

Characteristic	1	2^*c*^	3^*d*^	4^*e*^	5^*f*^	6^*g*^
Cell size (µm)	1.5–2.5 × 0.6–0.7	3.0–6.0 × 0.5–0.7	2.0–3.0 × 0.8–1.0	2.0–6.0 × 0.4	2.0–5.0 × 0.3–0.6	2.5–3.5 × 0.6–0.8
Colony pigmentation	White	Grayish pink	Beige	Slightly brownish/orange	ND	Creamy white
Motility	+	+	−	+	+	+
NaCl for growth (% [wt/vol])						
Range	0–9	3–17	<6	0–10	0–20	0–21
Optimum	0–3	10–15	ND	0.5–4	5–10	3
pH for growth						
Range	6.0–9.0	7.0–9.0	6.5–9.5	7.0–10.0	6.5–8.5	6.5–10.0
Optimum	7.0–8.0	8.0	7.0–9.0	ND	7.5	7.0–9.5
Temperature for growth (°C)						
Range	20–50	10–52	ND	15–45	10–50	15–42
Optimum	30–37	25–30	30	42	37	30
Oxidase	+	w	w	+	w	w
Catalase	+	+	+	+	+	+
Polar lipids	DPG, PG, PE, APL	DPG, PG, PL and others	DPG, PG, PL, APL	DPG, PG, PL, APL	DPG, PG, PL	ND
Major fatty acids (>10%)	Anteiso-C_15:0_, iso-C_15:0_, iso-C_14:0_, C_16:0_, iso-C_16:0_	Anteiso-C_15:0_, anteiso-C_17:0_, iso-C_15:0_, iso-C_16:0_	Anteiso-C_15:0_, iso-C_15:0_, anteiso-C_17:0_	iso-C_15:0_, anteiso-C_15:0_, anteiso-C_17:0_	anteiso-C_15:0_, iso-C_15:0_	iso-C_14:0_, iso-C_16:0_, iso-C_15:0_, anteiso-C_15:0_
Predominant menaquinone	MK-7	MK-7	MK-7	MK-7	MK-7	MK-7
Genome size (bp)	2,730,537	4,064,678	3,389,443	3,542,177	3,324,657	3,630,528
DNA G+C content (mol%)	42.68	35	38.0	36	37.0	35

^*a*^ Strains:1, 44XB^T^; 2, *Ornithinibacillus salinisoli* CGMCC 1.15809^T^; 3, *Ornithinibacillus contaminans* DSM 22953^T^; 4, *Ornithinibacillus bavariensis* DSM 15681^T^; 5, *Oceanobacillus halotolerans* CGMCC 1.17002^T^; 6, *Oceanobacillus iheyensis* JCM 11309^T^.

^*b*^ +, positive; −, negative; w, weakly positive; ND, no data available; DPG, diphosphatidylglycerol; PG, phosphatidylglycerol; PE, phosphatidylethanolamine; APL, unidentified aminophospholipid; PL, unidentified phospholipid.

^*c*^ Data for *Ornithinibacillus salinisoli* CGMCC 1.15809^T^ were obtained from reference 17 and this study.

^*d*^ Data for *Ornithinibacillus contaminans* DSM 22953^T^ were obtained from reference 18 and this study.

^*e*^ Data for *Ornithinibacillus bavariensis* DSM 15681^T^ were obtained from reference 19 and this study.

^*f*^ Data for *Oceanobacillus halotolerans* CGMCC 1.17002^T^ were obtained from reference 20 and this study.

^*g*^ Data for *Oceanobacillus iheyensis* JCM 11309^T^ were obtained from reference 21 and this study.

### Genome, phylogenetic analyses, and ecological distribution of 44XB^T^

16S rRNA gene sequencing revealed that strain 44XB^T^ exhibited the highest similarities with *Ornithinibacillus salinisoli* LCB256^T^ (96.36%), *Ornithinibacillus contaminans* CCUG 53201^T^ (96.17%), *Virgibacillus marseillensis* Marseille-P3610^T^ (95.75%), and *Oceanobacillus profundus* CL-MP28^T^ (95.57%). These identity results were lower than the 16S rRNA gene sequence identity threshold for prokaryotic species delineation (98.65%), suggesting that 44XB^T^ was a novel species (22). Phylogenetic analysis using 16S rRNA gene sequences demonstrated that 44XB^T^ formed a clade with *Oceanobacillus halotolerans* YIM98839^T^ but was distinguished from the genera *Ornithinibacillus*, *Lentibacillus*, *Virgibacillus*, and *Amphibacillus* (Fig. 1D and E). The neighbor-joining (NJ) and maximum-likelihood (ML) phylogenetic trees exhibited similar topologies although the bootstrap values for the branches formed by 44XB^T^ and *Oceanobacillus halotolerans* YIM98839^T^ were relatively low (Fig. 1D and E). Consequently, assigning 44XB^T^ to any known taxon at the genus level was difficult if relying solely on 16S rRNA gene sequences.

**Fig 2:**
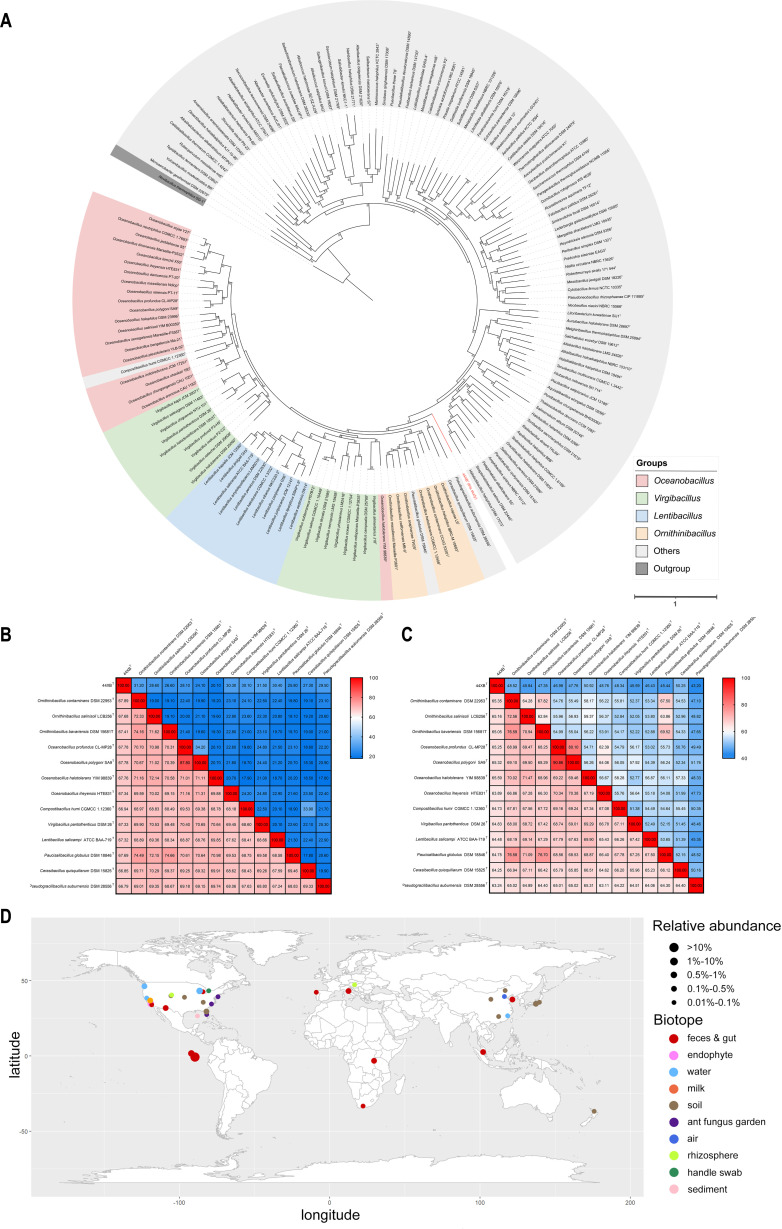
Genome-based phylogenetic tree, OGRIs, and global ecological distribution of *A. chroicocephali* 44XB^T^. (**A**) The maximum-likelihood genome-based phylogenetic tree of strain 44XB^T^. The colors represent different taxonomic groups. The tree was rooted using *Novibacillus thermophilus* SG 1^T^ as an outgroup. The numbers on the nodes indicate the number of UBCG genes (92 in total) supporting this topology, with the numbers exceeding 46 (greater than 50%) displayed on the nodes. Bar, 1 substitution per nucleotide position. (**B**) Heatmap with dDDH and ANI results; upper right: dDDH values, lower left: ANI values. (**C**) Heatmap with AAI and POCP results; upper right: POCP values, lower left: AAI values. (**D**) Global distribution of *A. chroicocephali* based on 75 16S rRNA gene amplicon data sets with a relative abundance of strain 44XB^T^ of at least 0.01%. Detailed information for each 16S rRNA gene amplicon data set can be found in Table S5. Some data sets have similar coordinates, meaning that some data points may overlap, particularly in the data sets from the Galápagos Islands. The world map was generated from the R package “maps” v3.4.1 using the R package “ggplot2” v3.4.2 in R v4.1.2.

The whole genome of 44XB^T^ was composed of a single circular chromosomal DNA of 2.73 Mbp (2,730,537 bp, 410 × coverage), and the G+C content was 42.68 mol% (Fig. S3). The genome contained 72 tRNA genes, 8 5S rRNA genes, 9 16S rRNA genes, 9 23S rRNA genes, and 2,754 predicted genes, accounting for 86.21% of the whole-genome length. In the phylogenetic tree reconstructed using the Up-to-date Bacterial Core Genes (UBCG) pipeline, strain 44XB^T^ formed an independent clade between the genera *Oceanobacillus*, *Ornithinibacillus*, *Virgibacillus*, *Lentibacillus,* and other genera within the *Bacillaceae* (Fig. 2A). Thus, 44XB^T^ was parallel to other related genera. Furthermore, although *Oceanobacillus halotolerans* YIM98839^T^ formed a monophyletic clade with 44XB^T^ in the 16S rRNA phylogenetic tree, a degree of evolutionary divergence was present with strain 44XB^T^ in the whole-genome phylogenetic tree, suggesting that strain 44XB^T^ might represent a novel genus.

Overall genomic relatedness indices (OGRIs) have been widely used in the discovery and delineation of new species and genera. Strain 44XB^T^ showed the highest digital DNA‒DNA hybridization (dDDH) and average nucleotide identity (ANI) values of 31.50% and 67.89%, respectively, among the closely related strains, which were significantly below the species demarcation criteria of 70% and 95%–96% for dDDH and ANI, respectively (Fig. 2B) (23, 24). Furthermore, strain 44XB^T^ exhibited a maximum percentage of conserved proteins (POCP) value of 50.92% with closely related strains, which slightly exceeded the threshold of 50% for genus delimitation proposed by Qin et al. (Fig. 2C) (25). Notably, the 50% POCP threshold for genus delimitation is considered overly conservative in the family *Bacillaceae* (26, 27). As observed in the heatmap, the POCP values among the genera *Oceanobacillus*, *Compostibacillus*, *Virgibacillus*, *Lentibacillus*, *Paucisalibacillus*, and *Cerasibacillus* generally exceeded the 50% threshold (Fig. 2C). Therefore, the relatively smaller POCP values indicated that 44XB^T^ was significantly differentiated from its related genera. Finally, the average amino acid identity (AAI) values were between 63.24% and 65.05% for 44XB^T^ and the type species of closely related genera, which were in the range of 60%–80% AAI values for distinguishing related but different genera and the 65%–70 % AAI values for distinguishing genera in the family *Bacillaceae* (Fig. 2C) (28–30). Thus, based on the differences in phenotypic, physiological, chemotaxonomic, OGRI, and phylogenetic characteristics, and OGRIs, strain 44XB^T^ was determined to represent a novel genus and species, named *Aciduricibacillus chroicocephali*.

We investigated the global ecological distribution of *A. chroicocephali* to determine its potential ecological significance. The complete 16S rRNA sequence of strain 44XB^T^ was submitted to the Integrated Microbial Next Generation Sequencing (IMNGS) platform with a sequence similarity cutoff of 99% (31). Of the 500,048 16S rRNA gene amplicon data sets, 242 data sets contained sequences with ≥99% similarity to 44XB^T^, accounting for 0.048% of the total. This novel isolate was mainly distributed in soil and host-associated samples (including feces, cloacal swabs, and animal bodies), and the abundance levels in host-related samples were significantly higher than those in soil samples (Fig. 2D; Fig. S4A; Table S5). A substantial number of data sets were obtained from bird feces and cloacal swabs, and we observed that 44XB^T^ was concentrated within the population of Darwin’s finches on the Galápagos Islands (Fig. S4B). This ecological distribution might indicate that strain 44XB^T^ was a normal component of the gut microbiotas of some birds.

### Metabolic characteristics and multicopy urate oxidase of 44XB^T^

**Fig 3:**
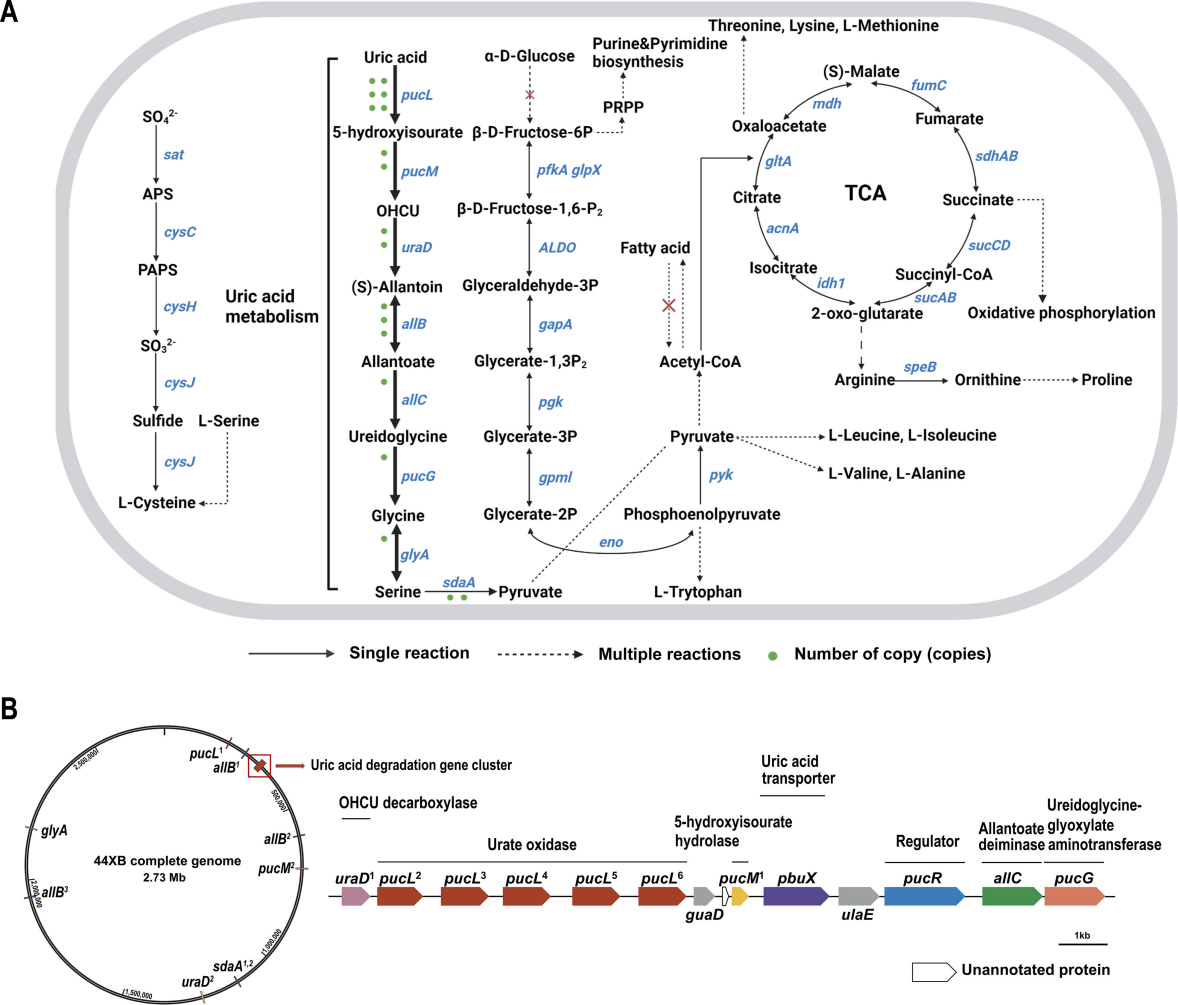
Uric acid metabolic reconstruction of *A. chroicocephali* 44XB^T^. (**A**) Metabolic reconstruction of the uric acid-utilizing pathway of strain 44XB^T^. The dashed arrows indicate reaction pathways involving multiple steps, while the solid arrows indicate the reactions involving a single step catalyzed by the annotated enzyme. Red cross marks in the dashed arrows indicate that the reactions could not be completed because of the lack of key enzymes. Green points show the number of gene copy/copies. (**B**) Genomic locations of the genes in the uric acid metabolic pathway and the uric acid degradation gene cluster. The numbers following the genes denote the sequential numbering of multiple copies of the gene.

Strain 44XB^T^ showed a limited substrate utilization spectrum in our carbon source utilization tests; among all tested substrates, only uric acid and allantoin supported the growth of 44XB^T^. We reconstructed its metabolic pathway according to the annotations (Fig. 3A). 44XB^T^ possessed an incomplete glycolytic pathway, lacking the genes encoding hexokinase (EC 2.7.1.1), glucokinase (EC 2.7.1.2), and glucose-6-phosphate isomerase (EC 5.3.1.9), which might contribute to the difficulty in utilizing some types of sugars. In addition, the fatty acid biosynthesis pathway was found in the genome, but the fatty acid degradation pathway was missing. Metabolic reconstruction identified the complete uric acid metabolic pathway in the 44XB^T^ genome. In this pathway, uric acid is sequentially converted to allantoin, serine, and pyruvate and then enters the tricarboxylic acid cycle for oxidative phosphorylation and the biosynthesis of key metabolites, including amino acids, nucleosides, fatty acids, and teichoic acid. Notably, the genes encoding the enzymes involved in the conversion of uric acid to allantoate (*pucL*, *pucM*, *uraD*, and *allB*) were present in multiple copies. We illustrated the genomic locations of the genes in the uric acid metabolic pathway and identified a new uric acid degradation gene cluster (Fig. 3B). A total of 6 *pucL* gene encoding urate oxidase (EC 1.7.3.3), which catalyzes the conversion of uric acid to 5-hydroxyisourate, were found in the genome. Of these, five of the copies were located in the uric acid degradation gene cluster, and the remaining *pucL* copy was located upstream. Multiple sequence alignment and phylogenetic analyses showed that these genes were homologous but not perfectly identical, sharing 70.42%–99.70% amino acid identity with each other (Fig. S5A). This was the first report of the presence of tandem urate oxidase genes. To exclude any sequencing errors and test the genes stability, we resequenced the whole genome of 44XB^T^ after consecutive subculturing 20 times, and the sequencing results revealed that the *pucL* copies remained stable (Fig. S5B). We then used transcriptome sequencing to analyze the expression of genes in the uric acid metabolic pathway (Fig. S5C). Transcription of 44XB^T^ *pucL* genes was notably active, with all six *pucL* genes being transcribed, of which numbers 1, 2, 4, 5, and 6 were dominant. Taking these results together, the uric acid-related metabolic characteristics and ecological distribution of 44XB^T^ suggest that it might play a role in uric acid metabolism in the gut.

### Strain 44XB^T^ could ameliorate hyperuricemia

**Fig 4:**
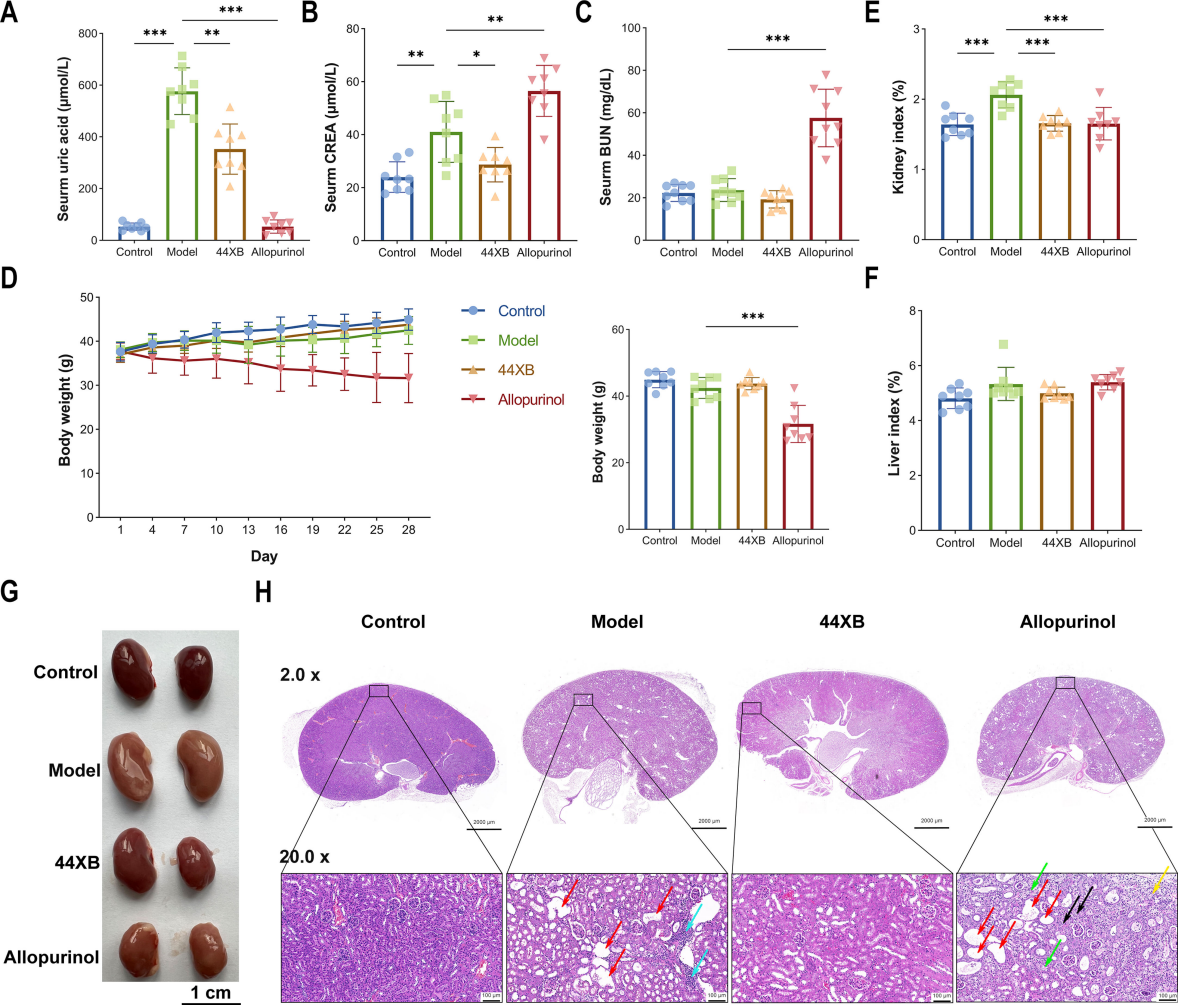
*A. chroicocephali* 44XB^T^ had the ability to alleviate hyperuricemia in mice. (**A**) The levels of serum uric acid after modeling for 4 weeks. (**B**) The levels of serum creatinine (CREA) after modeling for 4 weeks. (**C**) The levels of serum blood urea nitrogen (BUN) after modeling for 4 weeks. (**D**) Bodyweight changes throughout 4 weeks (*n* = 8) and the final bodyweights on the last day of the experiment. (**E**) Kidney indexes in each group (kidney weight/body weight). (**F**) Liver indexes in each group (liver weight/body weight). (**G**) Kidney morphologies in each group. (**H**) Hematoxylin and eosin (H&E)-stained images of the kidneys in each group (bars, 2,000 µm and 100 µm at 2.0× and 20.0×, respectively). Red arrows, tubular dilatation; blue arrows, lymphocyte infiltration; green arrows, renal tubular epithelium degeneration; black arrows, eosinophilic cast; yellow arrows, fibrosis. Bars show the mean ± SD. *P < 0.05; **P < 0.01; ***P < 0.001.

We studied the *in vivo* effects of 44XB^T^ in hyperuricemic mice. We first assessed the influence of potassium oxonate (a urate oxidase inhibitor used in modeling) on the growth of 44XB^T^, and the results showed that 44XB^T^ was able to grow under the pressure of 100 mM potassium oxonate and retained nearly 40% urate oxidase activity (intracellular enzyme activity), suggesting that the influence of potassium oxonate was considered acceptable. We constructed a hyperuricemic mouse model using hypoxanthine and potassium oxonate to evaluate the *in vivo* effects of 44XB^T^. The results showed that the serum uric acid levels in the model group were significantly increased compared with those in the control group (from an average of 53.1 to 576.6 µmol/L, *P* < 0.001). In the 44XB group, gavage with strain 44XB^T^ significantly reduced the serum uric acid levels compared with those in the model group, from an average of 567.7 µmol/L to 352.9 µmol/L (*P* < 0.01). In the positive control group (allopurinol group), allopurinol reduced serum uric acid levels to a level similar to those in the control group (Fig. 4A). Notably, the serum creatinine levels in the model group were significantly higher than those in the control group (*P* < 0.01), whereas the serum creatinine levels were significantly reduced after treatment with 44XB^T^ (Fig. 4B). We observed that the serum creatinine and urea nitrogen levels were significantly elevated (*P* < 0.01) in the allopurinol group compared with those in the control and model groups (Fig. 4B and C), consistent with the previous study (32). The combination of hypoxanthine and potassium oxonate had minor adverse effects on body weight, though no significant body weight change was observed in the 44XB group, indicating that 44XB^T^ was relatively safe (Fig. 4D). There were no significant differences in the liver indices (liver weight/body weight) among the four groups, but the kidney index (kidney weight/body weight) of the model group was significantly higher than that of the control group, while in the 44XB group, it was significantly decreased (*P* < 0.001, Fig. 4E and F).

We focused on the effects of 44XB^T^ intervention on the kidney. The kidneys in the model group were swollen, while the kidneys in the 44XB group showed no appreciable differences from those in the control group (Fig. 4G). However, in the allopurinol group, the morphology of the kidneys was abnormal, and their surfaces were uneven, which exhibited common kidney toxicity. H&E staining of the model group revealed partial tubular dilation, flattened epithelial cells, and occasional lymphocyte infiltration, which were significantly ameliorated by treatment with 44XB^T^; in the allopurinol group, kidney injury became more severe (Fig. 4H). Proinflammatory cytokines, including IL-1β, TNF-α, and IL-6, are mediators of kidney injury, and the downregulation of inflammatory cytokines supported strain 44XB^T^-induced alleviation of kidney damage (Fig. S6A). Regarding the effects on the gut, the expression levels of IL-6 and TNF-α were not significantly different among the groups, and 44XB^T^ downregulated IL-1β expression (Fig. S6B). In addition, the expression levels of the tight junction proteins ZO-1 and Occludin were not different among the four groups (Fig. S6C). These results indicated that strain 44XB^T^ could reduce serum uric acid levels and alleviate kidney damage in hyperuricemic mice, and no apparent adverse events caused by the strain were observed.

### The mechanism by which strain 44XB^T^ functions in the treatment of hyperuricemia

**Fig 5:**
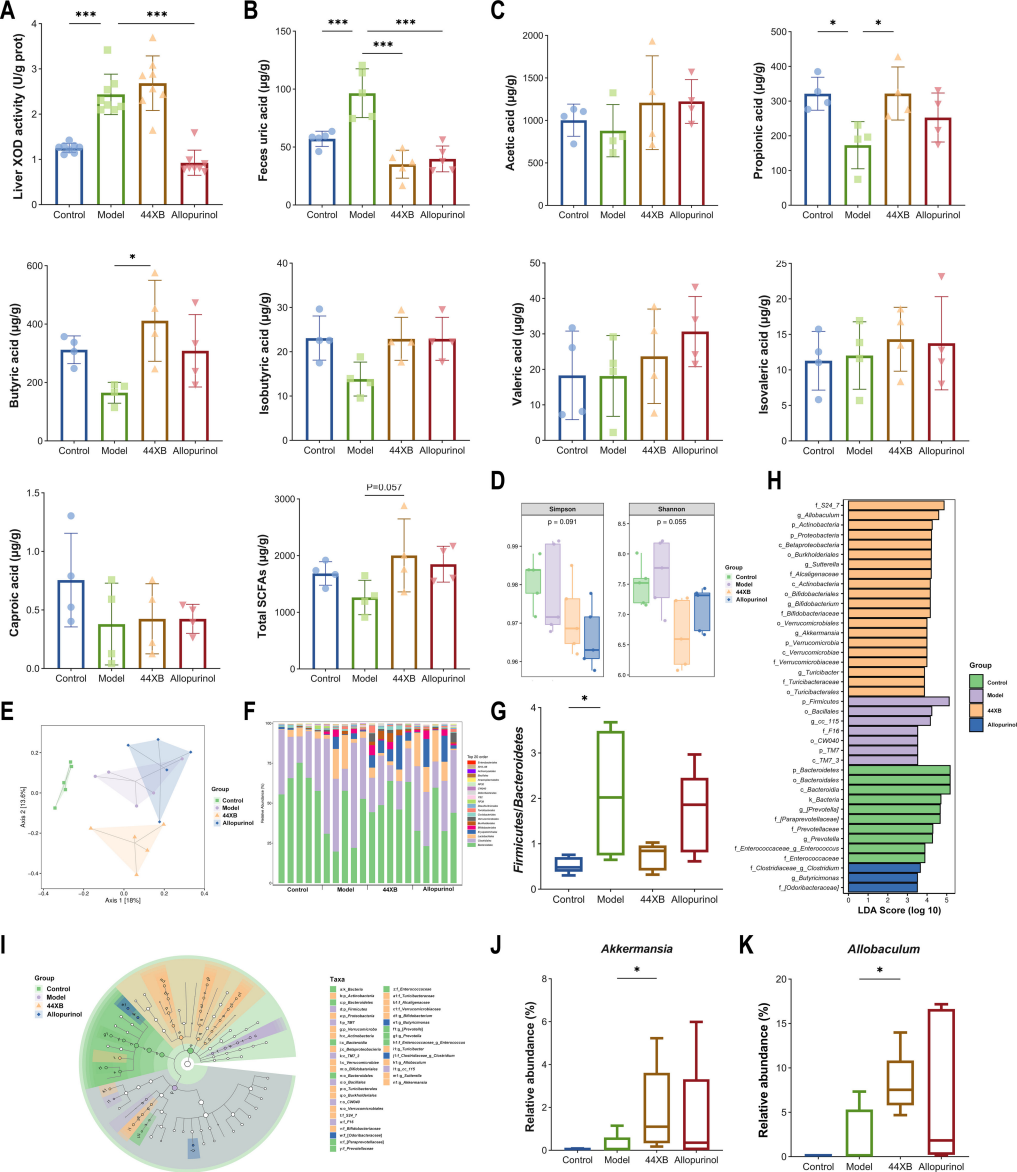
*A. chroicocephali* 44XB^T^ promoted uric acid degradation, remodeled gut microbiota and elevated SCFA production. (A) Effect on XOD in the liver. (B) Uric acid content in feces. (C) SCFA concentrations in the colonic contents. (D) α-Diversity indexes: Simpson and Shannon indexes. (E) PCoA plot of Bray‒Curtis dissimilarity indexes. (F) The abundance of microbiota at the order level. (G) The ratio of Firmicutes/Bacteroidetes. (H, I) Taxa with significant differences identified by LEfSe. (J, K) The relative abundances of *Akkermansia* and *Allobaculum*. Bars show the mean ± SD. *P < 0.05; **P < 0.01; ***P < 0.001.

We further studied the mechanism by which strain 44XB^T^ intervenes in hyperuricemia. Intragastric administration of hypoxanthine increased the availability of the substrate of uric acid synthesis. The activity of xanthine oxidase (XOD) in the liver was greatly increased in the model and 44XB groups, while administration of allopurinol, a competitive xanthine oxidase inhibitor, significantly decreased the liver XOD activity in the allopurinol group (Fig. 5A). Moreover, we analyzed uric acid levels in feces and found that the uric acid concentrations in the model group were significantly higher than those in the control group and were reduced in the 44XB group (Fig. 5B) .To explain this phenomenon, we analyzed short-chain fatty acids (SCFAs) and microbiota in the colonic contents. The concentrations of propionic acid in the colonic contents of the model group were significantly lower than the control group, and the administration of 44XB^T^ significantly increased the concentrations of propionic acid and butyric acid (Fig. 5C). In addition, the total SCFAs of the 44XB group were increased compared with that of the model group (*P* = 0.057). Alpha diversity was evaluated by the Shannon and Simpson diversity indices and was reduced in the 44XB group without a significant difference(Fig. 5D). In principal coordinate analysis (PCoA), the gut microbiota was changed in hyperuricemic mice, while 44XB^T^ remodeled the gut microbiota (Fig. 5E). We analyzed the composition of the microbiota at the phylum and genus levels. The strain 44XB^T^ reversed the changes in the *Firmicutes*/*Bacteroidetes* ratio, and the relative abundances and differential taxon were shown (Fig. 5F through I). Finally, 44XB^T^ specifically increased the abundances of *Akkermansia* and *Allobaculum*, which were butyrate/propionate-producing bacteria (Fig. 5J and K). In conclusion, strain 44XB^T^ have positive therapeutic effects on hyperuricemia by promoting uric acid degradation, remodeling the gut microbiota and elevating the production of SCFAs.

## DISCUSSION

Similar to higher primates, including humans, birds do not express active urate oxidase, and uric acid is the final product of their purine metabolism (33). However, birds have a unique uric acid excretion pathway. Highly concentrated urine with a large amount of uric acid is excreted into the cloaca through two ureteral openings, and then uric acid can be retrogradely transported into the hindgut and cecum which possess abundant and diverse microbiota. Consequently, the bird gut is a potential source of uric acid-degrading bacteria (34). Although progress in uric acid-degrading bacteria isolated from birds was made in recent years, few studies have focused on the bacteria themselves and their subsequent applications (35, 36). In this study, we isolated a uric acid-degrading strain 44XB^T^ from seagull feces and identified it as a novel genus within the family *Bacillaceae* using polyphasic taxonomic approaches. The particular physiological and biochemical characteristics of 44XB^T^ deserve special attention. 44XB^T^ can only utilize uric acid and allantoin as its sole carbon and nitrogen sources and growth in complex media without uric acid is difficult to achieve, which are unusual. A small genome may exhibit specific nutrient requirements, and the genome of 44XB^T^ is 2.73 Mbp, which is the smallest among the related genera (37). This may be because the happen of widespread gene loss events, result in the ability to utilize some sugars, and fatty acids are deprived, as suggested by the incomplete glycolytic pathway and the missing fatty acid degradation pathway. The phenotypic characteristics associated with uric acid are very similar to those of *Metabacillus fastidiosus* (*Bacillus fastidiosus*), which also belongs to the family *Bacillaceae* (38, 39). Certain strains of *Metabacillus fastidiosus* exhibit scarce growth on complex media (e.g.*,* TSB and BHI media) and can also utilize uric acid and allantoin efficiently (39). Multiple copies of urate oxidase appear to be common in *Metabacillus fastidiosus*, and two copies of *pucL* (encoding urate oxidase) with lower sequence identity were found at separate locations in the genome of the extensively studied *Bacillus fastidiosus* ATCC 26904 (40). However, the presence of up to six copies of *pucL* and the tandem arrangement of the *pucL* genes in the genome make strain 44XB^T^ special. We identified a unique gene cluster containing five repetitive but different urate oxidase genes in the 44XB^T^ genome, and this was the first report of tandem urate oxidase genes. Six copies of urate oxidase may play a dosage compensatory role (as observed at the transcriptional level), allowing 44XB^T^ to grow rapidly in a medium with uric acid as the sole carbon source. We speculate that the urate oxidase produced by 44XB^T^ is a mixed enzyme. Differences among urate oxidase amino acid sequences may contribute to broadening the temperature and pH ranges of the enzyme-catalyzed reactions or enhancing heavy metal ion tolerance, thereby enabling 44XB^T^ to adapt to the original intestinal environment. Our subsequent studies will characterize the enzymatic characteristics of the six urate oxidases and explore the functional significance.

In the metabolic profiling, we observed that strain 44XB^T^ was uric acid-dependent and that growth was limited by the availability of uric acid, which was a feature conducive to the safety of *in vivo* experiments. Therefore, we validated the *in vivo* effects of 44XB^T^ in a hyperuricemia mouse model. The model mice administered 44XB^T^ exhibited lower serum levels of uric acid and creatinine and experienced attenuated kidney damage. Compared with allopurinol (allopurinol has found adverse effects on body weight and kidney damage) administration, the advantages of 44XB^T^ were the lower levels of less kidney damage and weight impact (32, 41). Existing research has demonstrated that some microbes have potential antihyperuricemic activities. *Lactobacillus gasseri* PA-3, *Lactobacillus rhamnosus* FHuBei281, *Lactobacillus rhamnosus* R31, and *Limosilactobacillus fermentum* JL-3 alleviate hyperuricemia by promoting purine degradation, restoring gut microbiota dysbiosis, inhibiting XOD activity, and promoting uric acid degradation, respectively (6, 42, 43). We observed a significant decrease in fecal uric acid in mice given 44XB^T^, suggesting that 44XB^T^ was directly or indirectly involved in the removal of uric acid *via* catalysis due to its multiple copies of urate oxidase. In addition, we found that the concentrations of propionic acid and butyric acid were significantly increased in the mice treated with 44XB^T^, and the total SCFAs tended to increase, possibly due to the alteration of the gut microbiota. Butyrate has been shown to inhibit proinflammatory cytokine production induced by monosodium urate crystals *in vitro*, and sodium butyrate can normalize serum uric acid levels in rats fed a high-fat diet, suggesting that the increase in butyrate induced by 44XB^T^ may be one of the mechanisms of action (44, 45). We also noted that the abundance of *Akkermansia muciniphila* was significantly increased in the 44XB group; *Akkermansia muciniphila* has been shown to attenuate hyperuricemia in mice, which may also contribute to the effects of 44XB^T^ (46). Strain 44XB^T^ reduced serum uric acid levels through gavage, with no evidence indicating its colonization in the mammalian gut, ensuring its biosafety. Collectively, we propose that the beneficial effects of 44XB^T^ on hyperuricemia can be ascribed to the combined effects of the unique urate oxidases and the remodeled gut microbiota, resulting in the degradation of uric acid and the production of SCFAs in the gut. These findings suggest that uric acid-degrading bacteria from bird guts may represent a new source for potential urate-lowering treatment strains.

Although our study raised many interesting findings, there were some limitations. First, we proposed the uric acid dependence phenomenon of strain 44XB^T^ and the underlying genomic and transcriptomic characteristics, but there was a lack of comprehensive analysis on all urate oxidases, including physiological and biochemical analyses. Additionally, the importance, mechanism, and evolutionary implications of tandem *pucL* with amino acid differences require further study. The in-depth study was expected to facilitate understanding of gene duplication and tandem duplication. In the follow-up studies, we plan to address these points and assess further applications of strain 44XB^T^, including comprehensive safety analyses for clinical application, and applications of *pucL* sequences for engineered (15) or lactic acid bacteria.

In conclusion, we isolated a uric acid-degrading bacterium from black-headed gull feces and named it *Aciduricibacillus chroicocephali* gen. nov., sp. nov. by employing polyphasic taxonomic approaches. Furthermore, we inferred its metabolic pathways, identified unique tandem urate oxidases, and verified its effect of alleviating hyperuricemia. Our findings verified the feasibility of utilizing bird gut uric acid-degrading bacteria for ULT and provided a potential model strain for studying bacterial uric acid metabolism and urate oxidase gene evolution.

### Taxonomy

#### Description of *Aciduricibacillus* gen. nov.

##### *Aciduricibacillus* (A.cid.u.ri.ci.ba.cil’lus. N.L. neut. n. acidum uricum, uric acid; L. masc. n. *bacillus*, a small rod; N.L. masc. n. *Aciduricibacillus*, a uric acid using rod)

The cells are gram-positive, strictly aerobic, rod-shaped, and motile. The major fatty acids (>10%) are anteiso-C_15:0_, iso-C_15:0_, iso-C_14:0_, C_16:0_, and iso-C_16:0_. The major polar lipids are phosphatidylglycerol (PG), diphosphatidylglycerol (DPG), phosphatidylethanolamine (PE), and three unidentified aminophospholipids (APLs). The major respiratory quinone is menaquinone-7 (MK-7). Uric acid is used as the sole source of carbon and nitrogen. The type species is *Aciduricibacillus chroicocephali*.

### Description of *Aciduricibacillus chroicocephali* sp. nov.

#### *chroicocephali* (chro.i.co.ce’pha.li. N.L. gen. n. *chroicocephali*, of the black-headed gull *Chroicocephalus ridibundus*)

Colonies are creamy-white, smooth, with white rings and are approximately 1–1.5 mm in diameter after incubation for 2 days at 37°C on UA medium. The strain cannot grow on LB medium, NA, BHI medium, nutrient agar, or blood agar. The strain is gram-positive, strictly aerobic, rod-shaped, motile, 0.6–0.7 µm wide and 1.5–2.5 µm long. Growth occurs in the presence of 0%–9% NaCl (optimum of 0%–3%), at 20–50°C (optimum at 30–37°C), and a pH of 6.0–9.0 (optimum of 7.0–8.0). The strain is positive for catalase and oxidase. In API ZYM, alkaline phosphatase, acid phosphatase, and esterase lipase (C8) were positive, whereas lipase (C14), valine arylamidase, cystine arylamidase, trypsin, α-chymotrypsin, α-galactosidase, β-galactosidase, β-glucuronidase, α-glucosidase, β-glucosidase, *N*-acetyl-β-glucosaminidase, α-mannosidase, and α-fucosidase were negative. No substrate is strongly utilized in the Biolog GENIII test. The strain can utilize uric acid and allantoin as its sole carbon and nitrogen sources, but not common carbon sources and amino acids, including glucose, pyruvate, serine, glycine, and among others. The strain can strongly utilize uric acid and produce urate oxidase. The DNA G+C content of the type strain is 42.68 mol% (by genome).

The type strain 44XB^T^ (=MCCC 1K08703^T^ = KCTC 43631^T^) was isolated from a seagull fecal sample collected from Qingdao Nanjiang Wharf, China. The GenBank accession numbers of the 16S rRNA and whole-genome sequences are OR125650 and CP129113, respectively.

## MATERIALS AND METHODS

### Bacterial isolation

Black-headed gull was identified by morphological characteristics before sample collection (47). The seagull feces used for isolation were collected from Qingdao Nanjiang Wharf (120°57′84″ N, 36°09′81″ E), and the samples were transferred to 1.5 mL Eppendorf tubes, placed on ice, and rapidly transported to the laboratory. Fecal samples were added to a 250-mL shake flask containing 100 mL of UA medium (5.0 g/L uric acid, 0.5 g/L yeast extract, 0.5 g/L NaCl, 0.5 g/L MgSO_4_·7H_2_O, 0.5 g/L K_2_HPO_4_, 2.0 g/L KH_2_PO_4_, pH 7.5) and cultured at 200 rpm and 37°C for 2 days (48). After enrichment, the culture solution was plated on UA agar medium (10^4^-fold dilution). The culture solution was inoculated into UA agar medium. After 3 days of cultivation, colonies that formed transparent zones were purified for multiple rounds, and pure cultures were stored in 20% (vol/vol) glycerol at −80°C. Full details on the media are provided in Table S7.

### Reference strains

The reference type strains *Ornithinibacillus salinisoli* CGMCC 1.15809^T^, *Ornithinibacillus contaminans* DSM 22953^T^, *Ornithinibacillus bavariensis* DSM 15681^T^, *Oceanobacillus halotolerans* CGMCC 1.17002^T^, and *Oceanobacillus iheyensis* JCM 11309^T^ were obtained from China General Microbiological Culture Collection Center (CGMCC), German Collection of Microorganisms and Cell Cultures (DSMZ, and Japan Collection of Microorganisms (JCM) (44–48).Information about these reference strains and 44XB^T^ is shown in Table 1; Table S8.

### Genomic DNA extraction, sequencing assembly, and annotation

Bacterial genomic DNA was extracted using a Bacteria Genomic DNA Extraction Kit (Takara, Japan). The concentration of genomic DNA was quantified using Qubit fluorometer (Thermo Fisher Scientific, USA), and agarose gel electrophoresis was used to confirm DNA integrity. The bacterial genome was sequenced using both the Nanopore and DNBSEQ platforms at BGI (Shenzhen, China). Canu (version 1.5) was used for subread correction and corrected reads assembly, followed by single-base correction using GATK software (49). Glimmer (version 3.02) and RNAmmer (version 1.2) were used to predict the genes and rRNAs, respectively (50, 51). Gene annotation was performed using the Clusters of Orthologous Groups (COG) and Kyoto Encyclopedia of Genes and Genomes (KEGG) databases (52, 53).

### Phylogenetic analyses

16S rRNA was amplified with the primer pair 27F and 1492R using Sanger sequencing (Sangon, China) (54). The 16S rRNA sequence similarity was evaluated in a BLAST search on the EzBiocloud server (https://www.ezbiocloud.net). To clarify the phylogenetic position of the novel isolate, 16S rRNA sequences from type strains belonging to the genera *Ornithinibacillus*, *Virgibacillus*, *Oceanobacillus*, *Lentibacillus*, and other related genera were downloaded from the GenBank and EzBiocloud databases. Then, 16S rRNA-based phylogenetic trees were reconstructed using NJ and ML methods in MEGA X (version 10.1.8) with Kimura’s two-parameter model and general time reversible model, respectively (55, 56). The topology of the trees was evaluated by bootstrapping 1000 iterations.

The genomes used for analyses were downloaded from the EzBiocloud and NCBI Assembly databases. The UBCG pipeline (version 3.0) was used to reconstruct a whole-genome-based phylogenomic tree, which contains 92 bacterial core genes and is widely used in phylogenetic analyses of novel genera and species (57, 58). During the reconstruction of the UBCG tree, Prodigal, Hmmsearch, MAFFT, and FastTree were used for gene prediction, identification of UBCG genes, sequence alignment, and ML tree reconstruction, respectively, and the tree was visualized by using the Interactive Tree Of Life (59–63).

### Overall genomic relatedness indices

The dDDH values were calculated using the Genome-to-Genome Digital Calculator (64). The ANI by BLAST values were computed using the Orthologous Average Nucleotide Identity Tool (65). POCP was calculated using a software package (https://github.com/2015qyliang/POCP), and AAI was generated from CompareM (https://github.com/donovan-h-parks/CompareM) (25).

### Phenotypic and biochemical characterization

Gram staining and spore staining kits (Solarbio, China) were used for stain-based identification protocols. Cells were fixed using an electron microscopy fixative (Servicebio, China) and observed using scanning electron microscopy (Hitachi, Japan) after dehydration. The motility of the novel lineage was assessed in semisolid UA medium containing 0.4% agar. Growth conditions, including temperature, pH, and NaCl tolerance, were assessed on UA medium. The growth temperature of the strain was tested at 10°C, 20°C, 30°C, 37°C, 45°C, 50°C, and 60°C. The growth at different pH values (4.0–11.0, at 1.0 pH unit intervals) was examined by adjusting the pH of the UA medium with a 0.1 M citric acid-citrate sodium buffer system (pH 3.0–5.0), a 0.1 M NaOH-KH_2_PO_4_ buffer system (pH 6.0–8.0), and a 0.1 M Na_2_CO_3_-NaHCO_3_ buffer system (pH 9.0–11.0) (66). NaCl tolerance was assessed across a range of NaCl concentrations (0, 1, 3, 5, 6, 7, 8, 9, 10, 15, and 20%, wt/vol). The anaerobic growth of the strain was tested by observing colony formation in an anaerobic jar with an anaerobic pack (MGC, Japan) after culturing for 7 days. Oxidase and catalase activities were assessed by adding an oxidase reagent (BioMérieux, France) and 3% hydrogen peroxide solution to fresh colonies. Carbon source use, enzyme activity, and other biochemical characteristics of the strains were determined using GENIII microplates (Biolog, USA) and API ZYM and API 20NE kits (BioMérieux, France). Urate oxidase activity was assayed using a Uricase Activity Assay Kit (Solarbio, China) by measuring the hydrogen peroxide released when uric acid was converted to allantoin. The sonication conditions were as follows: ultrasonic power of 150 W, with breaking for 3 s, cooling for 7 s, and continuing for 4 min. The protein concentration was detected using the BCA method, and the urate oxidase activity in the soluble fraction was calculated.

### Chemotaxonomic characterization

The polar lipids of the strains were analyzed using two-dimensional thin-layer chromatography (TLC) (67). Polar lipid samples were spotted onto a silica gel 60 F_254_ plate (Merck, Germany) after extraction and separated using a solvent system of chloroform: methanol: water (65:25:4, vol/vol/vol) in the first direction and chloroform: methanol: acetic acid: water (80:12:15:4, vol/vol/vol/vol) in the second direction. The total, sugar-containing, phosphorus-containing, and amino group-containing lipids were determined using molybdatophosphoric acid, α-naphthol/sulfuric acid, molybdenum blue, and ninhydrin, respectively. The whole-cell fatty acid composition was extracted using the standard MIDI protocol (Sherlock Microbial Identification System, version 6.0) and identified using gas chromatography (Agilent, USA) (68). Respiratory quinone was identified by UPLC-MS after TLC separation, as previously described (69).

### Transcriptome sequencing

Strain 44XB^T^ was cultured in UA basal medium (uric acid was the sole carbon and nitrogen sources) to OD_600_ = 0.6, and cells were collected for transcriptome sequencing at Allwegene (Beijing, China). RNA was extracted by the Trizol method (Invitrogen, USA) and treated with DNase I (Takara, Japan). RNA was quantified by Agilent 2100 Bioanalyzer (Agilent, USA), and the quality was assessed by agarose gel electrophoresis and NanoDrop spectrophotometer (Thermo Scientific, USA). Strand-specific libraries were constructed after a Ribo-off rRNA Depletion Kit V2 (Bacteria) (Vazyme, China) was used for removing rRNA. Library qualities were assessed on the Agilent Bioanalyzer 2100 system (Agilent, USA), followed by sequencing on the Novaseq 6000 platform (Illumina, USA), and the aired-end 150 bp reads were generated. Clean reads were mapped to the reference genome by Bowtie2 (version 2.2.6) after trimming the raw reads (70). HTSeq (version 0.5.4) was used to count the number of reads mapped to each gene, and the gene expression levels were estimated by Fragments Per Kilobase of transcript per Million mapped (FPKM) (71).

### Animals and experimental scheme

Thirty-two 5-week-old specific pathogen-free (SPF) male Kunming mice (KM mice) were purchased from Charles River (Beijing, China), housed in the experimental animal center of Qingdao University, and adaptively reared for 7 days. Mice were housed under SPF conditions at 23°C ± 2°C, 50% ± 10% relative humidity, and 12 h light and dark cycles. All mice were allowed free access to water and fed a standard rodent chow diet.

After 1 week of adaptation, the mice were divided into four groups. In model group, 44XB group, and allopurinol group, hypoxanthine (500 mg/kg; Aladdin, China) was administered by oral gavage, and potassium oxonate (200 mg/kg; Sigma‒Aldrich, USA) was administered by intraperitoneal injection 1 h after gavage (42, 72, 73). In control group, same volume of 0.5% CMC-Na solution was used. In addition to the control and model groups administered PBS, the 44XB group was administered 10^9^ colony-forming units of 44XB^T^, and the allopurinol group was administered an oral gavage of allopurinol (5 mg/kg; Solarbio, China) (72, 73). The treatment was given once a day for 4 weeks.

### Sample collection and biochemistry analysis

Feces were collected before the end of the experiment. At the end of the experiment, the mice were euthanized, and blood, liver, kidney, ileum, and colonic content samples were collected. Blood samples were centrifuged at 1,000 × *g* for 15 min to collect serum, and kidney samples were fixed with 4% paraformaldehyde and then processed for paraffin embedding. The concentrations of serum uric acid, creatinine, and blood urea nitrogen were determined using an automated chemistry analyzer (Rayto, China). Tissue samples were paraffin-embedded, and 4 µm sections were stained with hematoxylin-eosin (H&E). Digital slices were collected using an automatic digital slide scanner (3DHISTECH, Hungary). XOD activity was detected using a Xanthine Oxidase (XOD) Activity Assay Kit (Solarbio, China).

### Quantitative fluorescence PCR

The relative expression levels of inflammatory cytokines and tight junction proteins were detected by fluorescence quantitative PCR. Total RNA was extracted using a FastPure Cell/Tissue Total RNA Isolation Kit V2 (Vazyme, China). The RNA was reverse-transcribed using HiScript II Q RT SuperMix for qPCR (Vazyme, China), and the cDNA was quantitated using a NanoDrop One^C^ (Thermo Fisher Scientific, USA). The relative expression levels were quantified using AceQ qPCR SYBR Green Master Mix (Vazyme, China). The primers used are listed in Table S9.

### Determination of SCFAs and gut microbiota

The extraction and analysis of SCFAs were performed as previously described (74–76). Briefly, colonic content samples were mixed with 50 µL 15% phosphoric acid, 100 µL 125 µg/mL 4-methylvaleric acid, and 400 µL ether, and supernatants were collected after centrifugation. The supernatant analyses were performed using a TRACE 1300 gas chromatograph (Thermo Fisher Scientific, USA) lined to an ISQ 7000 single quadrupole mass spectrometer (Thermo Fisher Scientific, USA).

DNA was extracted using a QIAamp Fast DNA Stool Mini Kit (Qiagen, Germany), and the V3 and V4 hypervariable regions of the 16S rRNA gene were amplified using the primers 338F and 806R. PCR products were purified using VAHTS DNA Clean Beads (Vazyme, China), and the sequencing libraries were prepared using a TruSeq Nano DNA LT Library Prep Kit (Illumina, USA) after fluorometric quantitation. Next, libraries were sequenced on a NovaSeq instrument (Illumina, USA). Subsequent bioinformatic analysis was performed using QIIME 2.

### Statistical analysis

Statistical analysis was performed using GraphPad Prism version 8.0 and SPSS version 24. Data are presented as the mean ± standard deviation. One-way analysis of variance was used for data analysis, and *P* < 0.05 indicated significant differences (*P < 0.05, **P < 0.01, ***P < 0.001), while ns indicated no statistically significant difference.

## Data Availability

The 16S rRNA and whole-genome sequences of strain 44XB^T^ are available in NCBI GenBank under the accession numbers OR125650 and CP129113, respectively. Strain 44XB^T^ can be obtained from Marine Culture Collection of China (MCCC) and Korean Collection for Type Cultures (KCTC), and the type strain accession numbers are MCCC 1K08703T and KCTC 43631T.

## SUPPLEMENTAL MATERIAL

Supplemental figures and tables msphere.00025-24-s0001.docx Fig. S1 to S6; Tables S1 to S9.

ASM does not own the copyrights to Supplemental Material that may be linked to, or accessed through, an article. The authors have granted ASM a non-exclusive, world-wide license to publish the Supplemental Material files. Please contact the corresponding author directly for reuse.
