# Supplementary material for: Isolation and identification of uric acid-dependent Aciduricibacillus chroicocephali gen. nov., sp. nov. from seagull feces and implications for hyperuricemia treatment
Source: mSphere. 2024 May 30;9(6):e00025-24. doi: 10.1128/msphere.00025-24 (PMC11332149; doi:10.1128/msphere.00025-24)
Supplement: Supplemental figures and tables — Fig. S1 to S6; Tables S1 to S9. [file msphere.00025-24-s0001.docx]

Supplementary information

**
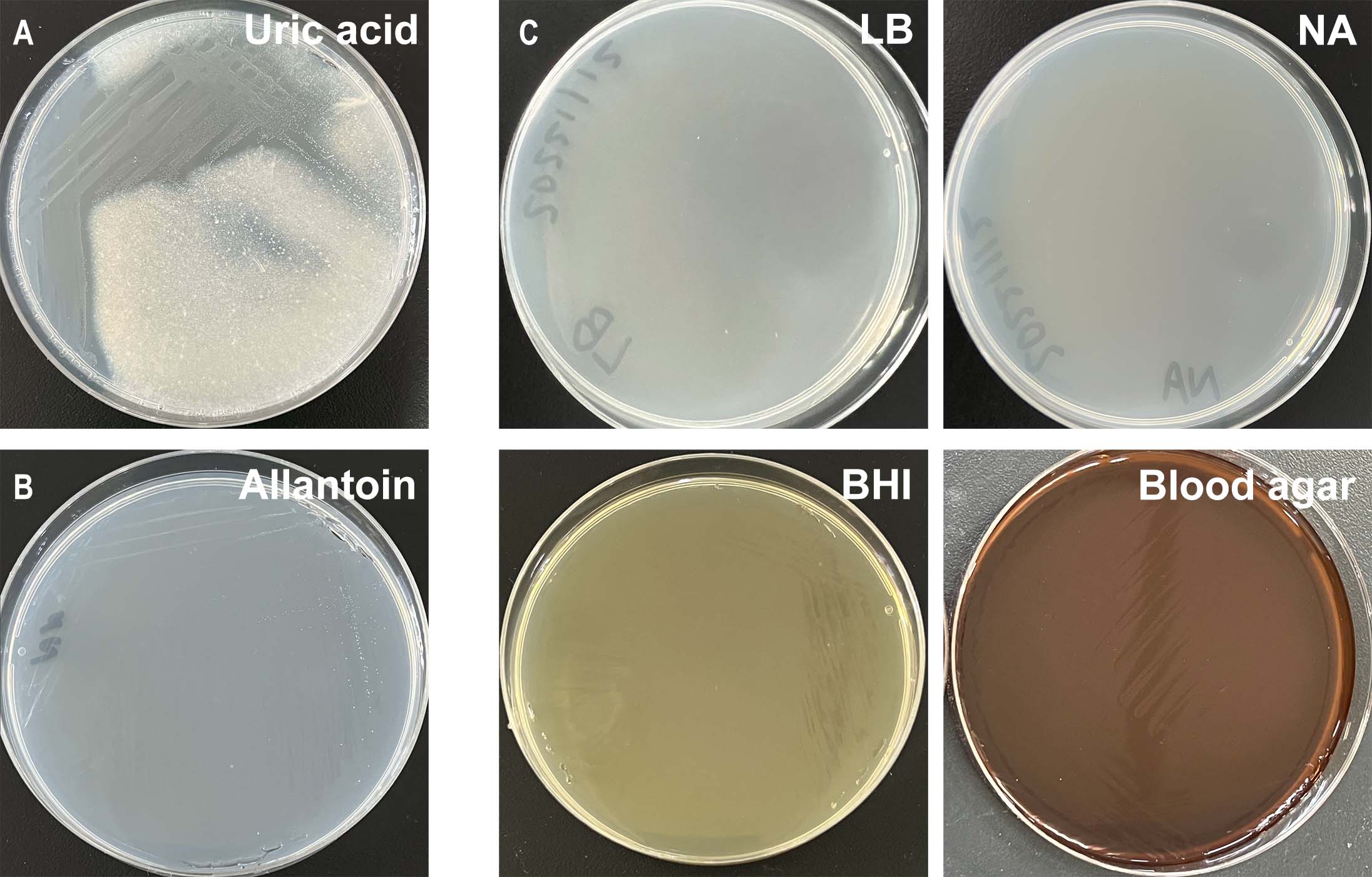
**

**Figure S1** Growth of *Aciduricibacillus chroicocephali* 44XB^T^ in different media. (A) Uric acid as the sole carbon and nitrogen source. (B) Allantoin as the sole carbon and nitrogen source. (C) Growth on LB, NA, BHI and blood agar media.

**
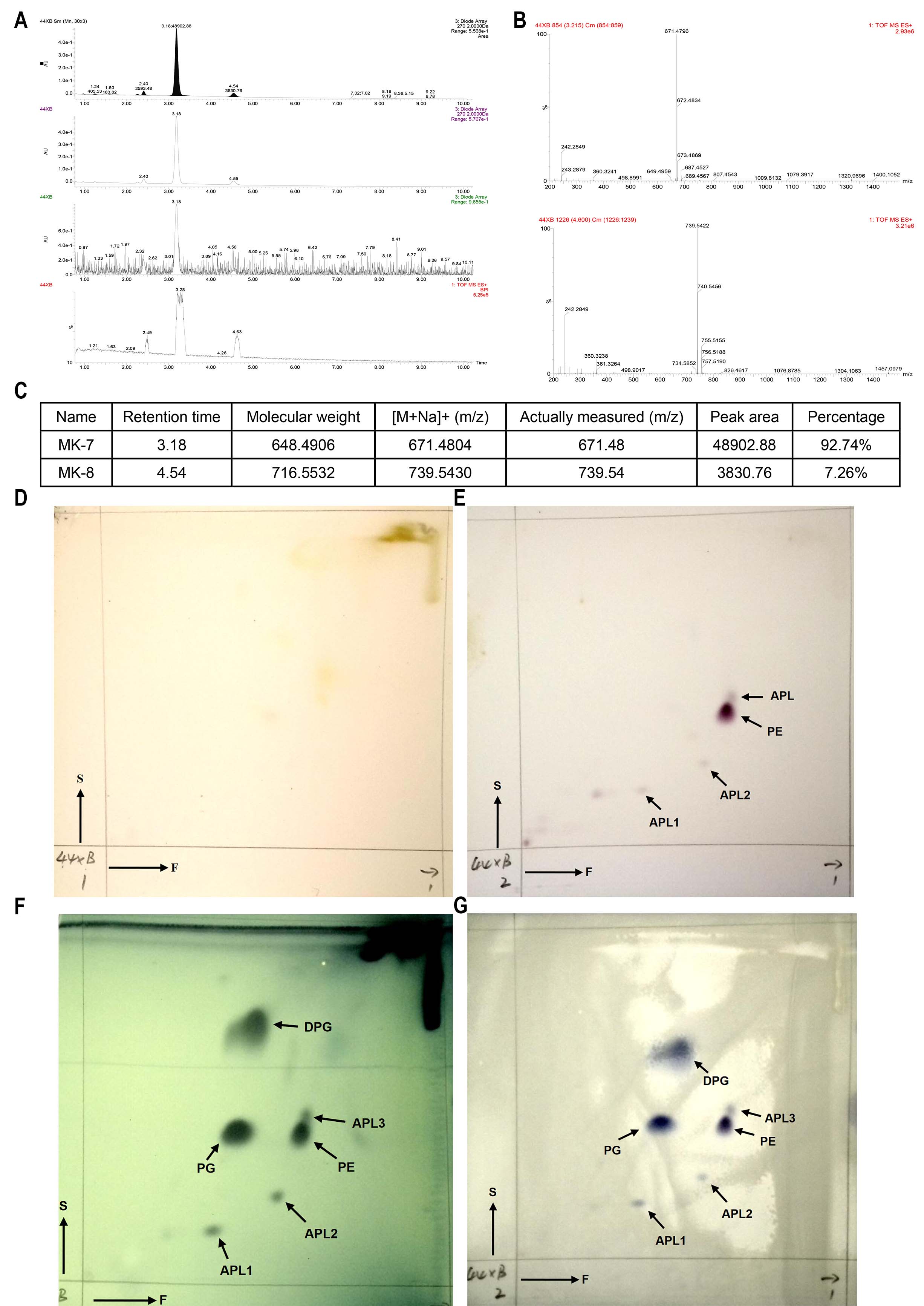
**

**Figure S2** Respiratory quinones and polar lipids of *A. chroicocephali* 44XB^T^. (A) UPLC chromatograms. (B) Mass spectrograms. (C) The major respiratory quinone was MK-7. (D) Glycolipid profile. (E) Aminolipid profile. (F) Total lipid profile. (G) Phospholipid profile. F: first dimension of TLC (chloroform-methanol-water 65:25:4, v/v/v); S: second dimension of TLC (chloroform-methanol-acetic acid-water 80:12:15:4, v/v/v/v). PG, phosphatidylglycerol; DPG, diphosphatidylglycerol; PE, phosphatidylethanolamine; APL, unidentified aminophospholipid.

**
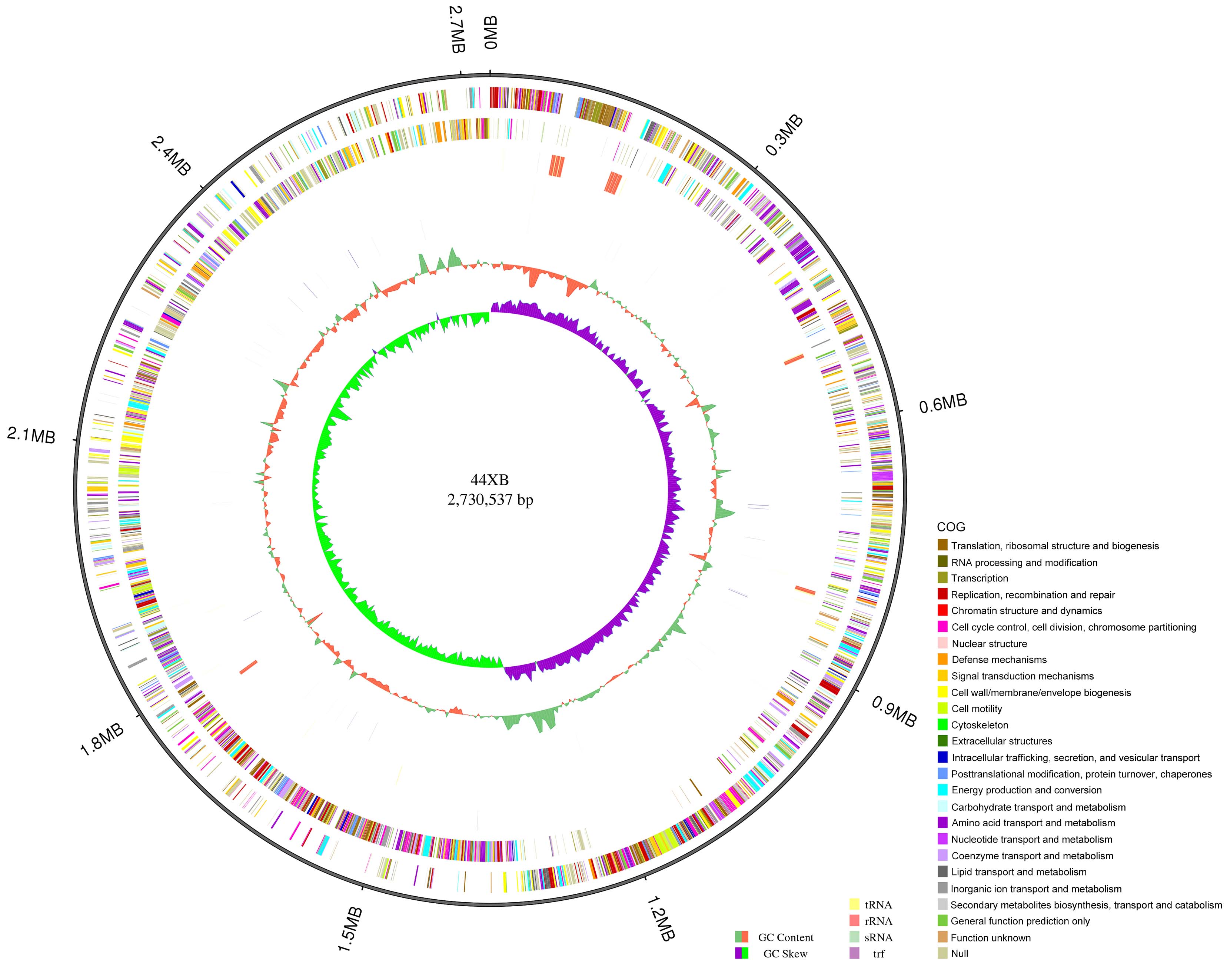
**

**Figure S3** Circular genomic map of *A. chroicocephali* 44XB^T^. From the outer to the inner rings: genome size scale, forward-strand genes, reverse-strand genes, forward-strand ncRNA genes, reverse-strand ncRNA genes, repeats, G+C content and GC skew. The genes are colored according to the Clusters of Orthologous Groups of proteins (COG) classification.

**
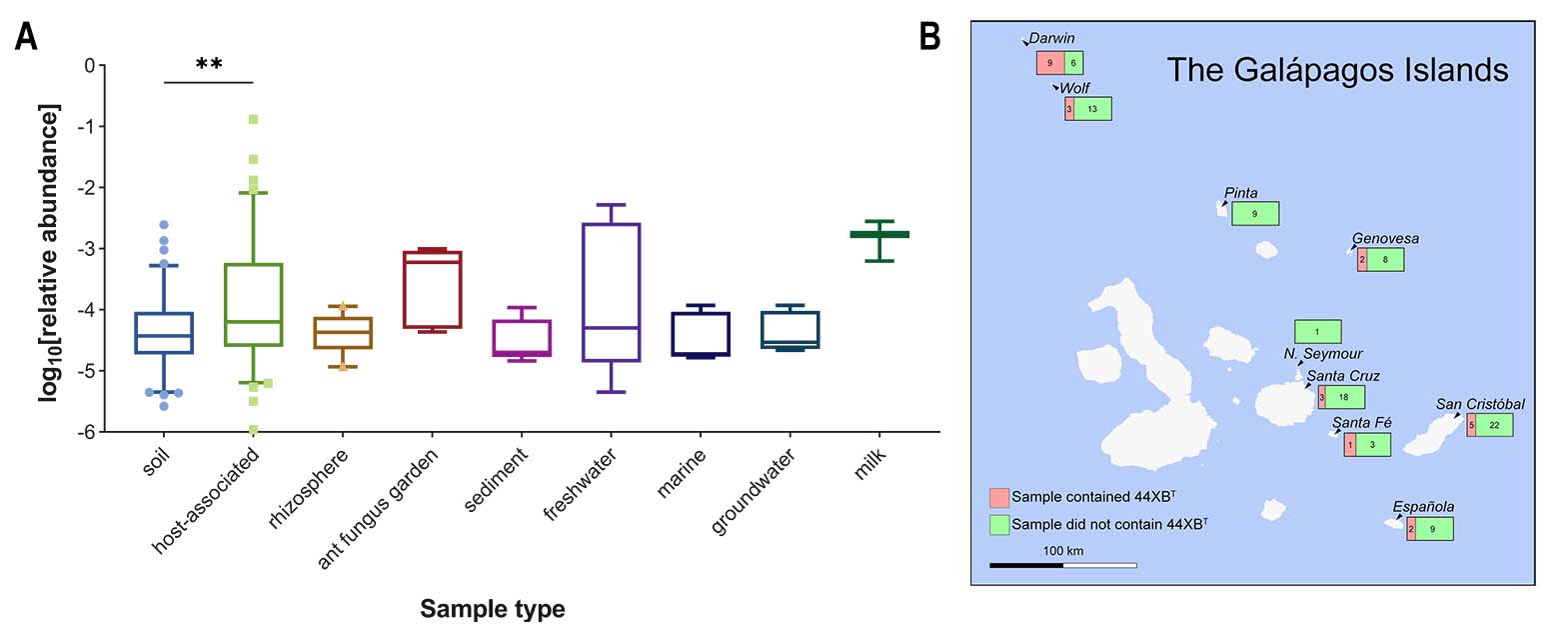
**

**Figure S4** Relative abundances in different biotopes and the detailed distribution on the Galápagos Islands. (A) Box plot of the relative abundances of *A. chroicocephali* across 9 major biotopes (n=219). The sample numbers for each biotope are as follows: soil: 86, host-associated: 85, rhizosphere: 21, ant fungus garden: 6, sediment: 6, freshwater: 5, marine: 4, groundwater: 4, and milk: 2. ***P* < 0.01. (B) Distribution of strain 44XB^T^ in Darwin's finches on Galápagos Islands.

**
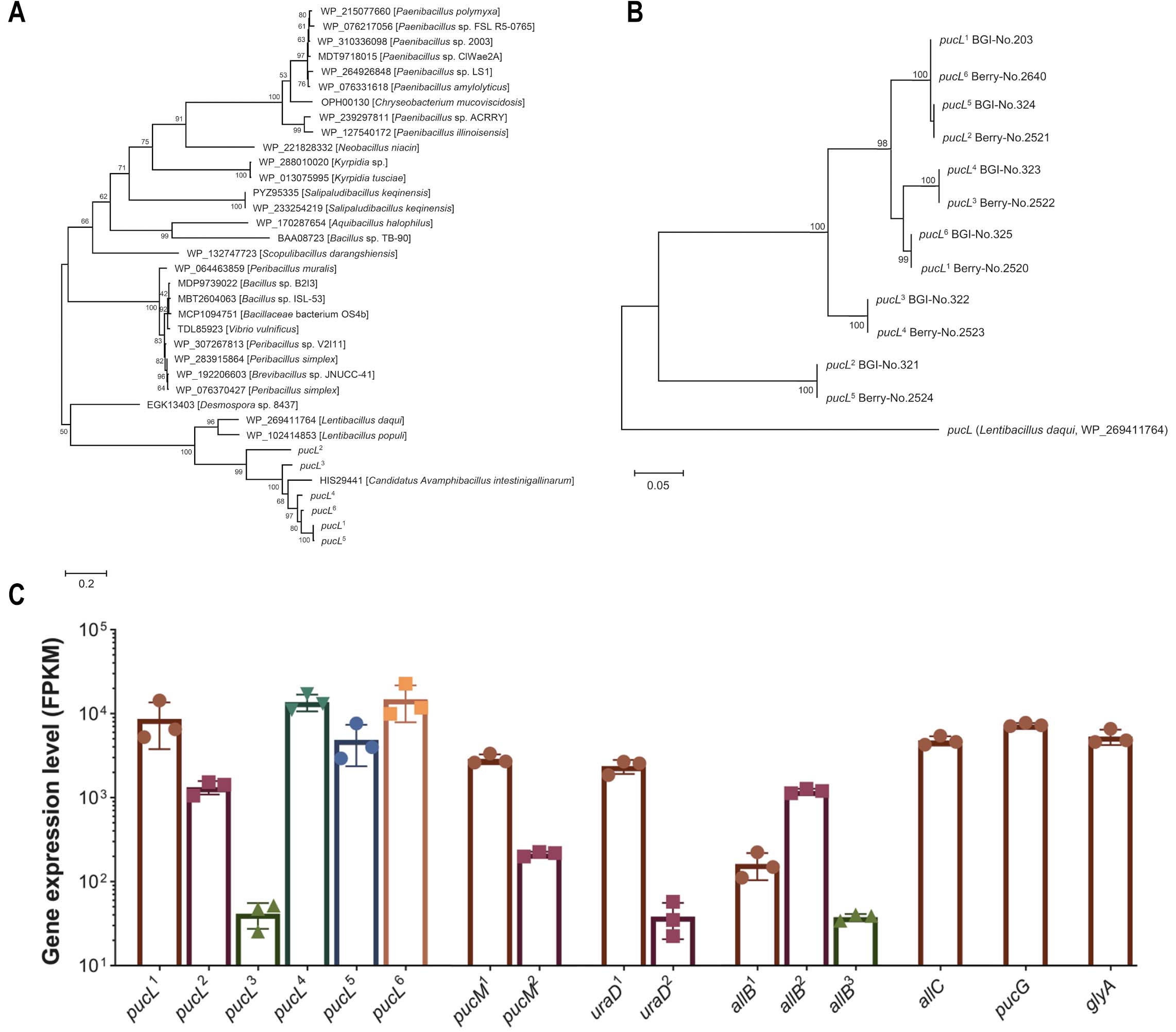
**

**Figure S5** Maximum likelihood phylogenetic tree based on the *pucL* amino acid sequences, and the expression of uric acid metabolism genes. (A) Maximum likelihood phylogenetic tree based on the *pucL* amino acid sequences. The number on *pucL* represents its location in the genome. Bootstrap values greater than 50% are shown at branch points. Bar, 0.2 substitutions per nucleotide position. (B) Maximum likelihood phylogenetic tree based on *pucL* nucleotide sequences obtained from two independent whole-genome sequences. BGI, Genomics Co. Ltd. (Shenzhen, China); Berry, Berry Genomics Co. Ltd. (Beijing, China); No., gene number. Bootstrap values greater than 50% are shown at branch points. Bar, 0.05 substitutions per nucleotide position. (C) Expression of uric acid metabolism genes in strain 44XB^T^.


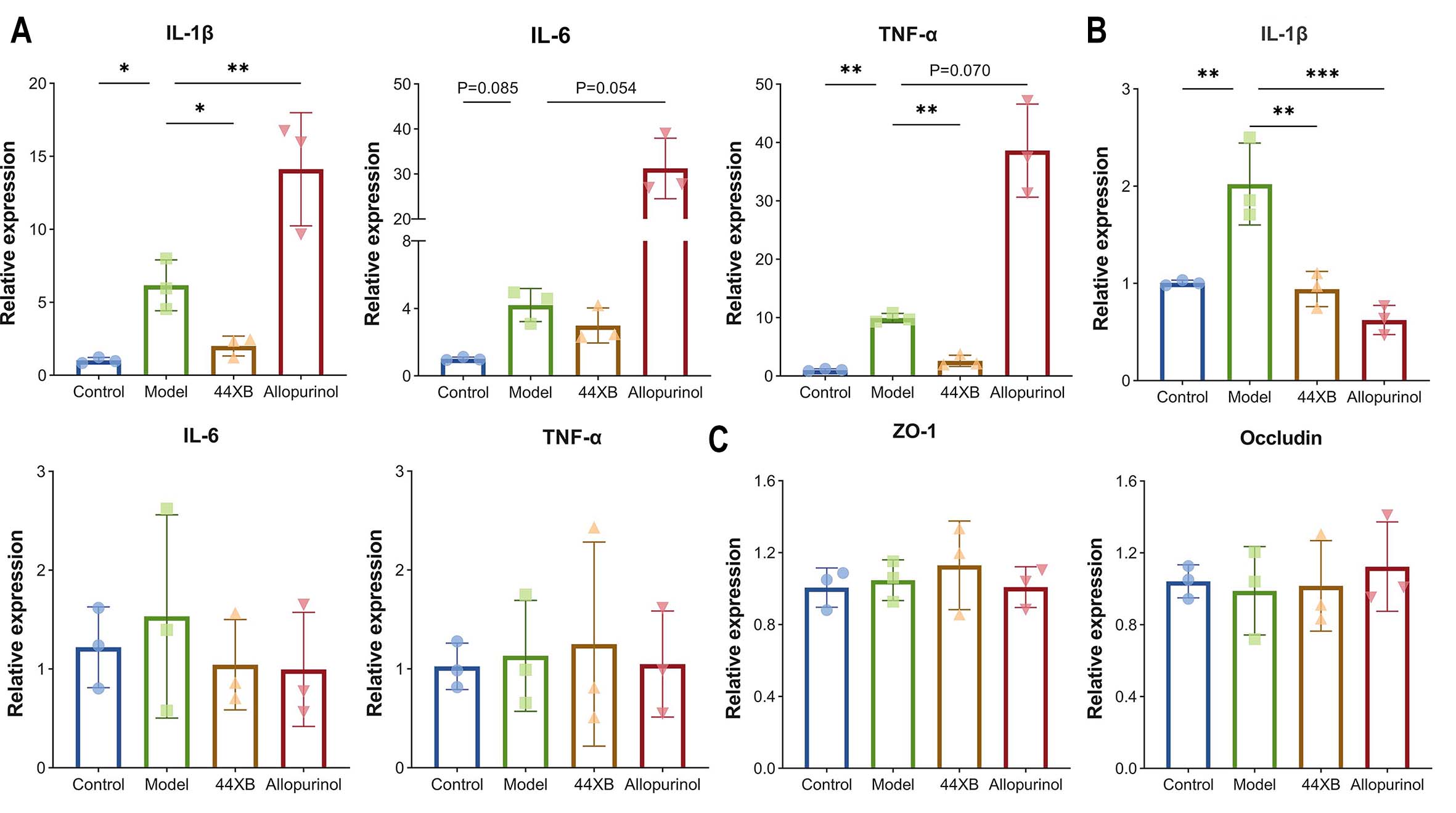


**Figure S6** Expression of inflammatory factors and tight junction proteins. (A) Expression of inflammatory factors in the kidney. Bars show the mean ± SD. (B) Expression of inflammatory factors in the ileum. (C) Expression of tight junction proteins in the ileum. Bars show the mean ± SD. **P* < 0.05; ***P* < 0.01; ****P* < 0.001.

**Table S1** Results of carbon source utilization tests of *A. chroicocephali* 44XB^T^.

| **Substrates** |  |
| --- | --- |
| **Uric acid** | **+** |
| **Allantoin** | **+** |
| **Glycine** | **-** |
| **L-serine** | **-** |
| **Pyruvate** | **-** |
| α-D-Glucose | - |
| D-Fructose | - |
| Sucrose | - |
| D-Galactose | - |
| D-Mannitol | - |
| Acetic acid | - |
| Lauric acid | - |
| Hexanoic acid | - |

*^a^*+, positive; -, negative.

*^b^*Substrates in the uric acid pathway were shown in bold.

**Table S2** Results of the API 20NE test of *A. chroicocephali* 44XB^T^ and the 5 reference strains.

| **Tests** | **Reactions/Enzymes** | **1** | **2** | **3** | **4** | **5** | **6** |
| --- | --- | --- | --- | --- | --- | --- | --- |
| NO_3_ | Reduction of nitrates to nitrites | - | - | - | - | + | - |
| NO_2_ | Reduction of nitrates to nitrogen | - | - | - | - | - | - |
| TRP | Indole production | - | - | - | - | - | - |
| GLU | Glucose fermentation | - | w | - | w | - | - |
| ADH | Arginine dihydrolase | - | - | - | - | - | - |
| URE | Urease | - | - | - | - | - | - |
| ESC | Esculin hydrolysis | w | + | + | + | + | + |
| GEL | Gelatin hydrolysis | - | + | + | - | w | + |
| PNPG | *β*-galactosidase | - | + | - | - | - | - |
| GLU | Glucose assimilation | - | - | w | - | - | + |
| ARA | Arabinose assimilation | - | - | - | - | - | + |
| MNE | Mannose assimilation | - | - | - | - | - | + |
| MAN | Mannitol assimilation | - | - | w | - | - | + |
| NAG | N-acetyl-glucosamine assimilation | - | - | w | - | - | + |
| MAL | Maltose assimilation | - | - | - | - | - | + |
| GNT | Potassium gluconate assimilation | - | - | w | - | - | + |
| CAP | Capric acid assimilation | - | - | - | - | - | - |
| ADI | Adipic acid assimilation | - | - | w | - | - | w |
| MLT | Malate assimilation | w | - | - | - | - | + |
| CIT | Trisodium citrate assimilation | - | - | w | - | - | + |
| PAC | Phenylacetic acid assimilation | - | - | - | - | - | - |

*^a^*Strains:1, 44XB^T^; 2, *Ornithinibacillus salinisoli* CGMCC 1.15809^T^; 3, *Ornithinibacillus contaminans* DSM 22953^T^; 4, *Ornithinibacillus bavariensis* DSM 15681^T^; 5, *Oceanobacillus halotolerans* CGMCC 1.17002^T^; 6, *Oceanobacillus iheyensis* JCM 11309^T^.

*^b^*+, positive; -, negative; w, weakly positive.

**Table S3** Results of the API ZYM test of *A. chroicocephali* 44XB^T^ and the 5 reference strains.

| **Enzymes** | **1** | **2** | **3** | **4** | **5** | **6** |
| --- | --- | --- | --- | --- | --- | --- |
| Water | - | - | - | - | - | - |
| Alkaline phosphatase | + | + | + | + | w | + |
| Esterase(C4) | w | + | + | + | + | + |
| Esterase lipase (C8) | + | + | + | + | + | + |
| Lipase (C14) | - | - | - | w | - | - |
| Leucine arylamidase | w | + | + | + | + | w |
| Valine arylamidase | - | - | w | - | w | - |
| Cystine arylamidase | - | - | - | - | + | w |
| Trypsin | - | - | w | w | + | w |
| *α*-chymotrypsin | - | + | + | + | + | + |
| Acid phosphatase | + | + | + | + | + | + |
| Naphtol-AS-BI-phosphohydrolase | w | + | w | w | w | w |
| *α*-galactosidase | - | - | - | - | - | - |
| *β*-galactosidase | - | - | - | - | - | - |
| *β*-glucuronidase | - | - | w | - | - | - |
| *α*-glucosidase | - | + | + | - | - | - |
| *β*-glucosidase | - | + | w | - | - | - |
| N-acetyl-*β*-glucosaminidase | - | - | - | - | - | - |
| *α*-mannosidase | - | - | - | - | - | - |
| *α*-fucosidase | - | - | - | - | - | - |

*^a^*Strains:1, 44XB^T^; 2, *Ornithinibacillus salinisoli* CGMCC 1.15809^T^; 3, *Ornithinibacillus contaminans* DSM 22953^T^; 4, *Ornithinibacillus bavariensis* DSM 15681^T^; 5, *Oceanobacillus halotolerans* CGMCC 1.17002^T^; 6, *Oceanobacillus iheyensis* JCM 11309^T^.

*^b^*+, positive; -, negative; w, weakly positive.

**Table S4** Results of the Biolog GENIII test of *A. chroicocephali* 44XB^T^ and the 5 reference strains.

| **Wells** | **Biolog GENIII substrates** | **1** | **2** | **3** | **4** | **5** | **6** |
| --- | --- | --- | --- | --- | --- | --- | --- |
| A1 | Negative Control | - | - | - | - | - | - |
| A2 | Dextrin | - | + | + | w | - | + |
| A3 | D-Maltose | - | + | + | w | + | + |
| A4 | D-Trehalose | - | + | + | + | + | + |
| A5 | D-Cellobiose | - | + | + | + | + | + |
| A6 | Gentiobiose | - | w | w | + | + | + |
| A7 | Sucrose | - | w | w | + | + | + |
| A8 | D-Turanose | - | w | w | w | + | + |
| A9 | Stachyose | - | w | w | w | + | + |
| A10 | Positive Control | + | + | + | + | + | + |
| A11 | pH 6 | w | + | + | + | + | + |
| A12 | pH 5 | w | - | - | - | - | - |
| B1 | D-Raffinose | - | w | - | w | - | + |
| B2 | *α*-D-Lactose | - | w | - | w | + | + |
| B3 | D-Melibiose | - | w | w | + | + | + |
| B4 | *β*-Methyl-D-Glucoside | - | w | + | w | + | + |
| B5 | D-Salicin | - | + | + | w | w | + |
| B6 | N-Acetyl-D-Glucosamine | - | + | + | + | + | + |
| B7 | N-Acetyl-*β*-D-Mannosamine | - | + | w | + | + | + |
| B8 | N-Acetyl-D-Galactosamine | - | - | - | - | + | + |
| B9 | N-Acetylneuraminic Acid | - | - | - | w | - | + |
| B10 | 1% NaCl | - | + | + | + | + | + |
| B11 | 4% NaCl | - | + | + | - | + | + |
| B12 | 8% NaCl | - | + | w | - | + | w |
| C1 | *α*-D-Glucose | - | + | + | + | w | + |
| C2 | D-Mannose | - | - | + | - | w | + |
| C3 | D-Fructose | - | + | + | + | + | + |
| C4 | D-Galactose | - | w | + | + | + | + |
| C5 | 3-Methyl Glucose | - | - | w | + | + | + |
| C6 | D-Fucose | - | w | w | + | + | + |
| C7 | L-Fucose | w | - | w | + | + | + |
| C8 | L-Rhamnose | - | - | w | + | + | + |
| C9 | Inosine | - | - | + | + | + | + |
| C10 | 1% Sodium Lactate | w | - | w | - | - | + |
| C11 | Fusidic Acid | - | - | - | - | + | - |
| C12 | D-Serine | w | + | + | + | + | - |
| D1 | D-Sorbitol | - | - | - | - | w | + |
| D2 | D-Mannitol | - | - | + | + | + | + |
| D3 | D-Arabitol | w | - | + | + | + | + |
| D4 | myo-Inositol | - | - | - | w | - | + |
| D5 | Glycerol | - | + | + | + | - | + |
| D6 | D-Glucose-6-PO4 | - | - | w | + | w | + |
| D7 | D-Fructose-6-PO4 | - | - | w | + | - | + |
| D8 | D-Aspartic Acid | - | - | w | + | + | + |
| D9 | D-Serine | - | w | - | + | - | + |
| D10 | Troleandomycin | - | + | + | + | + | w |
| D11 | Rifamycin SV | - | - | - | + | + | - |
| D12 | Minocycline | - | w | w | w | + | - |
| E1 | Gelatin | - | + | + | - | - | + |
| E2 | Glycyl-L-Proline | - | - | w | + | w | + |
| E3 | L-Alanine | - | + | + | + | + | + |
| E4 | L-Arginine | - | w | w | - | - | + |
| E5 | L-Aspartic Acid | w | - | w | + | - | + |
| E6 | L-Glutamic Acid | w | - | w | + | w | + |
| E7 | L-Histidine | w | - | + | + | + | + |
| E8 | L-Pyroglutamic Acid | w | - | w | + | w | + |
| E9 | L-Serine | - | + | w | + | w | + |
| E10 | Lincomycin | - | w | + | + | + | - |
| E11 | Guanidine HCl | - | - | - | w | - | - |
| E12 | Niaproof 4 | - | - | - | - | - | - |
| F1 | Pectin | - | - | - | - | - | + |
| F2 | D-Galacturonic Acid | - | - | w | + | - | + |
| F3 | L-Galactonic Acid Lactone | - | - | w | + | w | + |
| F4 | D-Gluconic Acid | - | - | - | - | - | + |
| F5 | D-Glucuronic Acid | - | - | - | w | w | + |
| F6 | Glucuronamide | w | - | - | w | + | + |
| F7 | Mucic Acid | - | - | w | w | - | + |
| F8 | Quinic Acid | w | - | w | + | w | + |
| F9 | D-Saccharic Acid | - | - | w | + | - | + |
| F10 | Vancomycin | - | - | - | - | + | - |
| F11 | Tetrazolium Violet | - | - | w | - | + | - |
| F12 | Tetrazolium Blue | - | - | - | - | - | - |
| G1 | *p*-Hydroxy-Phenylacetic Acid | - | - | - | w | - | + |
| G2 | Methyl Pyruvate | w | - | - | - | - | w |
| G3 | D-Lactic Acid Methyl Ester | - | w | + | + | w | + |
| G4 | L-Lactic Acid | - | w | - | w | - | + |
| G5 | Citric Acid | w | - | - | - | - | + |
| G6 | *α*-Keto-Glutaric Acid | - | w | + | + | + | + |
| G7 | D-Malic Acid | - | - | w | + | - | w |
| G8 | L-Malic Acid | - | w | w | + | + | + |
| G9 | Bromo-Succinic Acid | - | - | - | - | - | + |
| G10 | Nalidixic Acid | - | + | + | + | + | + |
| G11 | Lithium Chloride | w | + | + | + | + | + |
| G12 | Potassium Tellurite | w | + | + | - | - | w |
| H1 | Tween 40 | - | + | + | + | + | + |
| H2 | *γ*-Amino-Butyric Acid | - | - | - | w | - | + |
| H3 | *α*-Hydroxy-Butyric Acid | - | - | - | w | - | + |
| H4 | *β*-Hydroxy-D, LButyric Acid | - | - | w | + | - | + |
| H5 | *α*-Keto-Butyric Acid | - | + | - | - | w | + |
| H6 | Acetoacetic Acid | w | + | + | + | + | + |
| H7 | Propionic Acid | w | w | w | w | w | + |
| H8 | Acetic Acid | - | + | + | + | + | + |
| H9 | Formic Acid | - | - | - | w | - | w |
| H10 | Aztreonam | - | + | + | + | + | + |
| H11 | Sodium Butyrate | - | + | + | + | + | + |
| H12 | Sodium Bromate | - | - | - | - | - | - |

*^a^*Strains:1, 44XB^T^; 2, *Ornithinibacillus salinisoli* CGMCC 1.15809^T^; 3, *Ornithinibacillus contaminans* DSM 22953^T^; 4, *Ornithinibacillus bavariensis* DSM 15681^T^; 5, *Oceanobacillus halotolerans* CGMCC 1.17002^T^; 6, *Oceanobacillus iheyensis* JCM 11309^T^.

*^b^*+, positive; -, negative; w, weakly positive.

**Table S5** Cellular fatty acid profiles of *A. chroicocephali* 44XB^T^ and the 5 reference strains.

| **Fatty acids** | **1** | **2** | **3** | **4** | **5** | **6** |
| --- | --- | --- | --- | --- | --- | --- |
| C_14:0_ | 2.88 | TR | - | 1.26 | 8.51 | TR |
| C_16:0_ | **10.76** | 1.18 | TR | 5.00 | 2.46 | 1.66 |
| C_18:0_ | 2.76 | TR | TR | TR | TR | TR |
| iso-C_11:0_ | - | - | TR | - | 1.01 | - |
| iso-C_14:0_ | **11.34** | 6.75 | 3.13 | 1.02 | 8.51 | **25.26** |
| iso-C_15:0_ | **19.79** | **13.57** | **25.97** | **36.08** | **20.24** | **17.77** |
| iso-C_16:0_ | **10.5** | **12.14** | 4.89 | 2.49 | 6.01 | **19.93** |
| iso-C_17:0_ | 1.02 | 1.25 | 3.20 | 8.90 | TR | 1.43 |
| iso-C_17:1_ ω10*c* | TR | - | TR | 2.94 | - | TR |
| anteiso-C_13:0_ | TR | TR | TR | TR | 1.67 | TR |
| anteiso-C_15:0_ | **31.83** | **42.08** | **34.30** | **17.13** | **42.08** | **17.23** |
| anteiso-C_17:0_ | 2.61 | **14.80** | **17.44** | **13.15** | 5.92 | 2.98 |
| C_16:1_ ω7*c* alcohol | 1.23 | 3.39 | 2.97 | 0.80 | 3.57 | 6.14 |
| C_16:1_ ω11*c* | TR | TR | - | 2.00 | TR | TR |
| C_18:1_ ω9*c* | TR | - | TR | 1.02 | TR | TR |
| Summed Feature 4 | TR | TR | 2.42 | 1.95 | TR | TR |
| Summed Feature 8 | TR | TR | TR | 2.09 | TR | TR |

*^a^*Strains:1, 44XB^T^; 2, *Ornithinibacillus salinisoli* CGMCC 1.15809^T^; 3, *Ornithinibacillus contaminans* DSM 22953^T^; 4, *Ornithinibacillus bavariensis* DSM 15681^T^; 5, *Oceanobacillus halotolerans* CGMCC 1.17002^T^; 6, *Oceanobacillus iheyensis* JCM 11309^T^.

*^b^*TR, trace amount (<1%).

*^c^*Major fatty acids (>10%) were in bold.

*^d^*Summed features represented groups of fatty acids that cannot be separated using the MIDI system. Summed feature 4 contained iso-C_17:1_ I and/or anteiso/iso-C_17:1_ B. Summed feature 8 contained C_18:1_ ω7*c* and/or C_18:1_ ω6*c*.

**Table S6** Sample information of 16S rRNA gene amplicon datasets in which *A. chroicocephali* was presented.

| **SRA accession** | **Bioproject** | **Biosample** | **Organism** | **Sample type** | **Collect location** | **Relative abundance** |
| --- | --- | --- | --- | --- | --- | --- |
| SRR6486685 | PRJNA430731 | SAMN08378330 | gut metagenome | bird feces | 0.890678 S 89.609167 W | 0.13039 |
| SRR6486677 | PRJNA430731 | SAMN08378362 | gut metagenome | bird feces | 1.37389 S 89.720556 W | 0.02888 |
| SRR6413691 | PRJNA422103 | SAMN08162046 | human blood metagenome | peripheral blood | missing | 0.01326 |
| SRR6486796 | PRJNA430731 | SAMN08378267 | gut metagenome | bird feces | 1.67667 N 91.99889 W | 0.00931 |
| SRR6486767 | PRJNA430731 | SAMN08378265 | gut metagenome | bird feces | 1.67667 N 91.99889 W | 0.00541 |
| SRR8604851 | PRJNA497738 | SAMN10984369 | endophyte metagenome | pine needles | 34.81 N 119.14 W | 0.00540 |
| SRR8526270 | PRJNA366018 | SAMN06268066 | freshwater metagenome | freshwater | 43.1998 N 86.5698 W | 0.00524 |
| SRR8611111 | PRJNA497738 | SAMN10984369 | endophyte metagenome | pine needles | 34.81 N 119.14 W | 0.00488 |
| SRR6486713 | PRJNA430731 | SAMN08378236 | gut metagenome | bird feces | 1.39129 N 91.81917 W | 0.00418 |
| ERR1845509 | PRJEB13651 | SAMEA94555918 | milk metagenome | bovine milk | 36.7783 N 119.4179 W | 0.00279 |
| SRR8438009 | PRJNA502350 | SAMN10350831 | riverine metagenome | river water | 46.1812 N 123.1834 W | 0.00249 |
| DRR032976 | PRJDB3826 | SAMD00028753 | soil metagenome | farm soil | 34.642943 N 137.099529 E | 0.00246 |
| SRR8543610 | PRJNA612580;  PRJNA611257;  PRJNA520969 | SAMN10872800 | manure metagenome | chicken faces | Yantai | 0.00233 |
| SRR8543607 | PRJNA612580;  PRJNA611257;  PRJNA520969 | SAMN10872800 | manure metagenome | chicken faces | Yantai | 0.00190 |
| DRR018173 | PRJDB1856 | SAMD00002884 | gut metagenome | stomach contents | Lake Tanganyika | 0.00169 |
| SRR6486794 | PRJNA430731 | SAMN08378269 | gut metagenome | bird feces | 1.67667 N 91.99889 W | 0.00162 |
| SRR2530847 | PRJNA288822 | SAMN03852706 | soil metagenome | soil | 29.6899 N 81.9935 W | 0.00136 |
| SRR4000799 | PRJNA330953 | SAMN05442189 | gut metagenome | carnivore gut sample | 31.93 N 109.08 W | 0.00120 |
| SRR8543608 | PRJNA612580;  PRJNA611257;  PRJNA520969 | SAMN10872800 | manure metagenome | chicken faces | Yantai | 0.00117 |
| SRR1955317 | PRJNA280086 | SAMN03458928 | Gut microbiome | cloaca swab | 43.01 N 12.48 E | 0.00105 |
| SRR8705851 | PRJNA526361 | SAMN11096758 | snake metagenome | snake gut | 2.7305 N 102.0782 E | 0.00104 |
| SRR2907069 | PRJNA301166 | SAMN04240744 | gut metagenome | bird feces | Nuevo Leon | 0.00101 |
| SRR7519676 | PRJNA479679 | SAMN09601801 | ant metagenome | nesting material of ant | 27.62288 N 81.80935 W | 0.000993 |
| SRR3115558 | PRJNA305525 | SAMN04435474 | oil field metagenome | oil-containing produced water | Harvest Scovil Lake oil field | 0.000969 |
| SRR6486770 | PRJNA430731 | SAMN08378260 | gut metagenome | bird feces | 1.67667 N 91.99889 W | 0.000952 |
| SRR1740120 | PRJNA269635 | SAMN03256066 | soil metagenome | soil | 36.7226 S 175.877 E | 0.000951 |
| SRR7519692 | PRJNA479679 | SAMN09601773 | ant metagenome | nest of ant | 34.68167 N 78.59631 W | 0.000909 |
| SRR7519675 | PRJNA479679 | SAMN09601800 | ant metagenome | nest of ant | 27.62288 N 81.80935 W | 0.000800 |
| SRR6486745 | PRJNA430731 | SAMN08378254 | gut metagenome | bird feces | 1.67667 N 91.99889 W | 0.000707 |
| SRR4000770 | PRJNA330953 | SAMN05442321 | gut metagenome | multiplicata and bombifrons gut sample | 31.67 N 109.23 W | 0.000686 |
| SRR1955318 | PRJNA280086 | SAMN03458929 | gut metagenome | bird feces | 43.01 N 12.48 E | 0.000639 |
| ERR1841302 | PRJEB13651 | SAMEA92235418 | milk metagenome | milk | 36.7783 N  119.4179 W | 0.000626 |
| SRR8733618 | PRJNA527262 | SAMN11131699 | gut metagenome | fecal | missing | 0.000602 |
| SRR6486780 | PRJNA430731 | SAMN08378360 | gut metagenome | bird feces | 0.803056 S 90.044722 W | 0.000586 |
| SRR6486700 | PRJNA430731 | SAMN08378297 | gut metagenome | bird feces | 0.318056 N 89.95 W | 0.000584 |
| DRR032973 | PRJDB3826 | SAMD00028751 | soil metagenome | soil | 34.642943 N 137.099529 E | 0.000574 |
| SRR1956971 | PRJNA280270 | SAMN03460114 | gut metagenome | nest of ant | Panama: Gamboa | 0.000512 |
| SRR3179161 | PRJNA311763 | SAMN04496485 | soil metagenome | soil | 40.05372 N 105.64242 W | 0.000448 |
| SRR3213167 | PRJNA294696 | SAMN04040464 | invertebrate metagenome | cyclocephala sp. | 34.02 N 118.44 W | 0.000410 |
| SRR7519646 | PRJNA479679 | SAMN09601793 | ant metagenome | nest of ant | 39.42765 N 74.33865 W | 0.000394 |
| ERR584161 | PRJEB6978 | SAMEA2698692 | gut metagenome | ratsnake feces | 42.73254 N  84.55553 W | 0.000391 |
| DRR147233 | PRJDB7249 | SAMD00132343 | soil metagenome | soil | 35.7124361 N 139.407283 E | 0.000373 |
| SRR6486775 | PRJNA430731 | SAMN08378259 | gut metagenome | bird feces | 1.67667 N 91.99889 W | 0.000369 |
| SRR6486776 | PRJNA430731 | SAMN08378258 | gut metagenome | bird feces | 1.67667 N 91.99889 W | 0.000359 |
| ERR440286 | PRJEB5696 | SAMEA2377633 | metagenome | water | missing | 0.000326 |
| SRR3179193 | PRJNA311763 | SAMN04496517 | soil metagenome | soil | 39.10688 N 96.60914 W | 0.000303 |
| SRR2041154 | PRJNA284506 | SAMN03731650 | aquatic metagenome | water | 38.5539 N 121.7381 W | 0.000255 |
| SRR6486774 | PRJNA430731 | SAMN08378256 | gut metagenome | bird feces | 1.67667 N 91.99889 W | 0.000246 |
| SRR6486768 | PRJNA430731 | SAMN08378264 | gut metagenome | bird feces | 1.67667 N 91.99889 W | 0.000237 |
| ERR2780657 | PRJEB28512 | SAMEA4887027 | Struthio camelus | faeces | 33.35 S 22.12 E | 0.000219 |
| SRR4288900 | PRJNA343817 | SAMN05803456 | soil metagenome | soil | not applicable | 0.000214 |
| SRR6486696 | PRJNA430731 | SAMN08378294 | gut metagenome | bird feces | 0.318056 N 89.95 W | 0.000212 |
| SRR5853006 | PRJNA395135 | SAMN07372805 | gut metagenome | bird cloaca | 42.47 N 9.01 W | 0.000211 |
| SRR8494348 | PRJNA516815 | SAMN10821205 | gut metagenome | snake feces | missing | 0.000209 |
| ERR483043 | PRJEB6148 | SAMEA2471841 | soil metagenome | soil | 43.63 N 116.7 E | 0.000208 |
| SRR6486789 | PRJNA430731 | SAMN08378272 | gut metagenome | bird feces | 0.739111 S 90.301667 W | 0.000187 |
| SRR7641633 | PRJNA484211 | SAMN09762174 | soil metagenome | soil | 35.82 N 84.08 W | 0.000185 |
| SRR8664725 | PRJNA525335 | SAMN11047394 | air metagenome | air | 39.59 N 116.18 E | 0.000184 |
| ERR1095278 | PRJEB10962 | SAMEA3635652 | urban metagenome | outside door handle swab | 43.47 N 80.54 W | 0.000176 |
| SRR4288782 | PRJNA343817 | SAMN05803563 | soil metagenome | soil | not applicable | 0.000159 |
| SRR3292575 | PRJNA315524 | SAMN04565606 | freshwater metagenome | streamwater | 26.78 N 118.34 E | 0.000141 |
| SRR4288932 | PRJNA343817 | SAMN05803432 | soil metagenome | soil | not applicable | 0.000136 |
| SRR4288905 | PRJNA343817 | SAMN05803461 | soil metagenome | soil | not applicable | 0.000135 |
| SRR4288752 | PRJNA343817 | SAMN05803437 | soil metagenome | soil | not applicable | 0.000129 |
| SRR5155589 | PRJNA359373 | SAMN06197287 | soil metagenome | soil | 26.34 N 112.27 E | 0.000128 |
| SRR6486744 | PRJNA430731 | SAMN08378253 | gut metagenome | bird feces | 1.39129 N 91.81917 W | 0.000127 |
| SRR1811770 | PRJNA275598 | SAMN03350162 | soil metagenome | soil | Senegal | 0.000124 |
| SRR8361330 | PRJNA511010 | SAMN10621350 | marine sediment metagenome | sediment | 26.62 N 87.9 W | 0.000118 |
| SRR3722916 | PRJNA326072 | SAMN05256635 | plant metagenome | rhizosphere soil | 47.46 N 16.47 E | 0.000116 |
| SRR8648717 | PRJNA524467 | SAMN11026699 | rhizosphere metagenome | rhizosphere soil | not collected | 0.000115 |
| SRR6378139 | PRJNA422505 | SAMN08180796 | soil metagenome | soil | 37.708611 N 107.226944 E | 0.000114 |
| SRR2132524 | PRJNA286125 | SAMN03765173 | root metagenome | rhizosphere soil | 40.573461 N 105.080889 W | 0.000112 |
| SRR1123237 | PRJNA234104 | SAMN02581152 | sediment metagenome | sediment | missing | 0.000109 |
| SRR4288893 | PRJNA343817 | SAMN05803450 | soil metagenome | soil | not applicable | 0.000101 |
| SRR4000824 | PRJNA330953 | SAMN05442210 | gut metagenome | multiplicata and bombifrons gut microbiome | 31.67 N 109.23 W | 0.000101 |

**Table S7** Medium recipes.

| **Medium type** | **Medium recipe** |
| --- | --- |
| UA | 5.0 g uric acid, 0.5 g yeast extract, 0.5 g NaCl, 0.5 g MgSO_4_·7H_2_O, 0.5 g K_2_HPO_4_, 2.0 g KH_2_PO_4_, ddH_2_O up to 1.0 L, pH 7.5 |
| LB | 10 g peptone, 5.0 g yeast powder, 10.0 g NaCl, ddH_2_O up to 1.0 L, pH 7.2 |
| BHI | 10.0 g peptone, 12.5 g bovine brain leaching powder, 5.0 g bovine heart leaching powder, 5.0 g NaCl, 2.0 g glucose, 2.5 g Na_2_HPO_4_, ddH_2_O up to 1.0 L, pH 7.4 ± 0.2 |
| NA | 3.0 g beef extract, 10.0 g peptone, 5.0 g NaCl, ddH_2_O up to 1.0 L, pH 7.2 ± 0.1 |
| blood agar | 44.0 g Columbia blood agar base (Solarbio, China), ddH_2_O up to 1.0 L. After cooling to 55°C 80.0 mL defibrinated sheep blood was added |
| basal medium for carbon source tests | 0.2 g MgSO_4_·7H_2_O, 0.5 g NaH_2_PO_4_·H_2_O, 0.1 g CaCl_2_·2H_2_O, 0.5 g K_2_HPO_4_, ddH_2_O up to 1.0 L |

*^a^*1.5% agar was added for solid media.

*^b^*The concentrations of uric acid, allantoin, serine, and glycine were all 0.5%. The concentrations of sugars and sugar alcohols were 1%, and 2.0 g/L (NH_4_)_2_SO_4_ was added. The concentrations of other carbon sources were 0.2%, and 2.0 g/L (NH_4_)_2_SO_4_ was added.

**Table S8** Supplemental strains information.

| **Characteristic** | **1** | **2** | **3** | **4** | **5** | **6** |
| --- | --- | --- | --- | --- | --- | --- |
| Colony size | 1–1.5 mm | 1–2 mm | ND | 10 mm | ND | 2–3 mm |
| Colony color | Creamy white | Greyish pink | Beige | Slightly brownish/orange | ND | Creamy white |
| Colony shape | Circular | Circular | Circular | Circular | ND | Circular |
| Sample type | Black-headed gull feces | Saline-alkali soil | Human blood | Pasteurised milk | Hypersaline sediment | Deep sea sediment |
| Geographic location | Shandong Province | Gansu Province | Göteborg | Bavaria | Xinjiang Province | Okinawa |
| Country | China | China | Sweden | Germany | China | Japan |
| Reference | This study | (1) | (2) | (3) | (4) | (5) |

*^a^*Strains:1, 44XB^T^; 2, *Ornithinibacillus salinisoli* CGMCC 1.15809^T^; 3, *Ornithinibacillus contaminans* DSM 22953^T^; 4, *Ornithinibacillus bavariensis* DSM 15681^T^; 5, *Oceanobacillus halotolerans* CGMCC 1.17002^T^; 6, *Oceanobacillus iheyensis* JCM 11309^T^.

*^b^*Data was obtained from references and BacDive database (https://bacdive.dsmz.de/).

**Table S9** List of primers used in this study.

| **Primer name** | **Primer sequence 5'->3'** |
| --- | --- |
| 27F | AGAGTTTGATCMTGGCTCAG |
| 1492R | GGTTACCTTGTTACGACTT |
| 338F | ACTCCTACGGGAGGCAGCA |
| 806R | GGACTACHVGGGTWTCTAAT |
| GAPDH forward | GGTTGTCTCCTGCGACTTCA |
| GAPDH reverse | TGGTCCAGGGTTTCTTACTCC |
| IL-1β forward | CTCGCAGCAGCACATCAACAAG |
| IL-1β reverse | CCACGGGAAAGACACAGGTAGC |
| IL-6 forward | TACCACTTCACAAGTCGGAGGC |
| IL-6 reverse | CTGCAAGTGCATCATCGTTGTTC |
| TNF-α forward | GGTGCCTATGTCTCAGCCTCTT |
| TNF-α reverse | GCCATAGAACTGATGAGAGGGAG |
| ZO-1 forward | AACCCGAAACTGATGCTGTGGATAG |
| ZO-1 reverse | CGCCCTTGGAATGTATGTGGAGAG |
| Occludin forward | TGGCTATGGAGGCGGCTATGG |
| Occludin reverse | ACTAAGGAAGCGATGAAGCAGAAGG |

**References**

1. Gan L, Zhang H, Long X, Tian J, Wang Z, Zhang Y, Dai Y, Tian Y. 2018. *Ornithinibacillus salinisoli* sp. nov., a moderately halophilic bacterium isolated from a saline-alkali soil. Int J Syst Evol Microbiol 68:769–775.

2. Kämpfer P, Falsen E, Lodders N, Langer S, Busse HJ, Schumann P. 2010. *Ornithinibacillus contaminans* sp. nov., an endospore-forming species. Int J Syst Evol Microbiol 60:2930–2934.

3. Mayr R, Busse HJ, Worliczek HL, Ehling-Schulz M, Scherer S. 2006. *Ornithinibacillus* gen. nov., with the species *Ornithinibacillus bavariensis* sp. nov. and *Ornithinibacillus californiensis* sp. nov. Int J Syst Evol Microbiol 56:1383–1389.

4. Zhu WY, Yang L, Shi YJ, Mu CG, Wang Y, Kou YR, Yin M, Tang SK. 2020. *Oceanobacillus halotolerans* sp. nov., a bacterium isolated from salt lake in Xinjiang province, north-west China. Arch Microbiol 202:1545–1549.

5. Lu J, Nogi Y, Takami H. 2001. *Oceanobacillus iheyensis* gen. nov., sp. nov., a deep-sea extremely halotolerant and alkaliphilic species isolated from a depth of 1050 m on the Iheya Ridge. FEMS Microbiol Lett 205:291–297.
